# Identification of antiviral phytochemicals as a potential SARS-CoV-2 main protease (M^pro^) inhibitor using docking and molecular dynamics simulations

**DOI:** 10.1038/s41598-021-99165-4

**Published:** 2021-10-13

**Authors:** Chirag N. Patel, Siddhi P. Jani, Dharmesh G. Jaiswal, Sivakumar Prasanth Kumar, Naman Mangukia, Robin M. Parmar, Rakesh M. Rawal, Himanshu A. Pandya

**Affiliations:** 1 Department of Botany, Bioinformatics, and Climate Change Impacts Management, School of Sciences, Gujarat University, Ahmedabad, 380009 India; 2 BioInnovations, Bhayander (West), Mumbai, 401101 India; 3 Department of Zoology, School of Sciences, Gujarat University, Ahmedabad, 380009 India; 4 Department of Life Science, School of Sciences, Gujarat University, Ahmedabad, 380009 India

**Keywords:** Computational biology and bioinformatics, Molecular modelling

## Abstract

Novel SARS-CoV-2, an etiological factor of Coronavirus disease 2019 (COVID-19), poses a great challenge to the public health care system. Among other druggable targets of SARS-Cov-2, the main protease (M^pro^) is regarded as a prominent enzyme target for drug developments owing to its crucial role in virus replication and transcription. We pursued a computational investigation to identify M^pro^ inhibitors from a compiled library of natural compounds with proven antiviral activities using a hierarchical workflow of molecular docking, ADMET assessment, dynamic simulations and binding free-energy calculations. Five natural compounds, Withanosides V and VI, Racemosides A and B, and Shatavarin IX, obtained better binding affinity and attained stable interactions with M^pro^ key pocket residues. These intermolecular key interactions were also retained profoundly in the simulation trajectory of 100 ns time scale indicating tight receptor binding. Free energy calculations prioritized Withanosides V and VI as the top candidates that can act as effective SARS-CoV-2 M^pro^ inhibitors.

## Introduction

SARS-CoV-2 (severe acute respiratory syndrome coronavirus 2), the causative agent of Coronavirus disease (COVID-19), created havoc globally by infecting the large Human population and tremendously impacting the public health care system by its first outbreak in the form of pneumonia cluster in the Wuhan Province of China on 17/11/2019 and subsequent spread of COVID-19 worldwide^1,2^. The World Health Organization (WHO) declared the virus outbreak as a Public Health Emergency of International Concern on 30/01/2020 and a pandemic on 11/03/2020 to take aggressive measures to contain the spread of COVID-19 due to the alarming number of reported cases outside China by 13-fold (dated 3 August 2021, https://www.who.int/data)^3,4^. SARS-CoV-2 belongs to *Nidovirales* order of *Coronaviridae* family, a positive-strand and enclosed virus with RNA as the genetic material 78% similar to coronavirus noted for the SARS outbreak in the year 2003 (dated 3 August 2021, https://www.who.int/data)^5–7^. A series of earlier outbreak events such as SARS-CoV in 2002, H1N1 flu in 2009 and MERS-CoV in 2012 share the similar characteristics of developing viral fever affecting primarily the respiratory system leading to death^6,7^. COVID-19 can be detected in the early stage with various symptoms including viral pneumonia, dry cough, tiredness, taste or smell loss, difficulty or shortage of breath among others^8^.

Several research programs are being pursued to develop drug molecules targeting key protein targets of SARS-CoV-2. So far 12 coronavirus proteins have been characterized which can be categorized into two types, structural and non-structural proteins^9^. Main protease (M^pro^) is one such structural protein emerging as a promising target for antiviral drug development due to is role in viral replication and transcription^10–14^. Numerous drug discovery projects are currently pursued to identify potent inhibitors through the combination of computer-aided drug designing approaches and biochemical assays^15^. Structure-based virtual screening for identifying potential inhibitors from the collection of FDA-approved antiviral is extensively carried out to reduce the burden of designing new molecules and unknown fate in ADME/T properties and clinical trials^16–18^. Such repurposing of antivirals with known pharmacological effects are already being used in treating COVID-19 patients in emergency use (e.g., Remdesivir)^19^. Alamri et al. utilized molecular docking and dynamic simulations to identify M^pro^ candidates from Saudi medical plants^20^. Muhseen et al. used the above two methods to identify M^pro^ inhibitors from MPD3 phytochemical database^21–24^. Certain database-specific inhibitors search were also made including Super Natural II^25^, Marine Natural Product (MNP)^26^ and Traditional Chinese Medicine^27^. Fragment-based computational screening with constraints to target His45, Cys145 and His163 key residues identified a potent molecule with a triazole scaffold^28^. Balaramnavar et al*.* performed pharmacophore-based activity prediction of 66 FDA-approved drugs from different classes to identify imatinib molecule and experimentally verified the M^pro^ inhibition of 9.823 µM^29^.

Phytochemicals, the plant-derived molecules from medicinal plants (e.g., turmeric, Saif et al.^30^), act as a prominent reservoir for treating most of the viral infections documented from ethnobotanical applications and ancient literature on Ayurveda and traditional medicinal systems^31^. ul Qamar et al. identified an isoflavone from *Psorothamnus arborescens* with better binding affinity and key contacts with the catalytic dyad^21^. The chief phytochemical of *Asparagus racemosus,* Asparoside-C, exerted possible inhibitory action on the M^pro^ target by targeting the substrate-binding site^21^. Mengist et al. conducted a comprehensive review on the inhibitors design of M^pro^ target through in vitro and in silico approaches^32^. Remarkable progress have been made to design and develop new vaccines improving the immune response to SARS-CoV-2 and its select strains^33^. These include encapsulated RNA-based vaccine (mRNA-1273, Moderna)^34^, non-replicating viral vector (Sputnik V, Gamaleya; AZD1222, Oxford/AstraZeneca)^35,36^ and inactivated viral vaccine (Covaxin, Bharat Biotech)^37^. The evolution of new strains of SARS-Cov-2 (e.g., delta variant) poses a great obstacle in achieving desired immunization response to treat COVID-19^38^. Alternatively, drug development campaigns can be promoted to target intact structural proteins that may not undergo vast changes in its structure and its associated functions. In the present study, we performed a computational approach to identify M^pro^ inhibitors from a compiled library of antiviral small molecules^39,40^ using a hierarchical workflow of structure-based virtual screening, dynamic simulations and binding free energy calculations.

## Results and discussion

### Virtual screening of SARS-CoV-2 M^pro^ target

**Figure 1:**
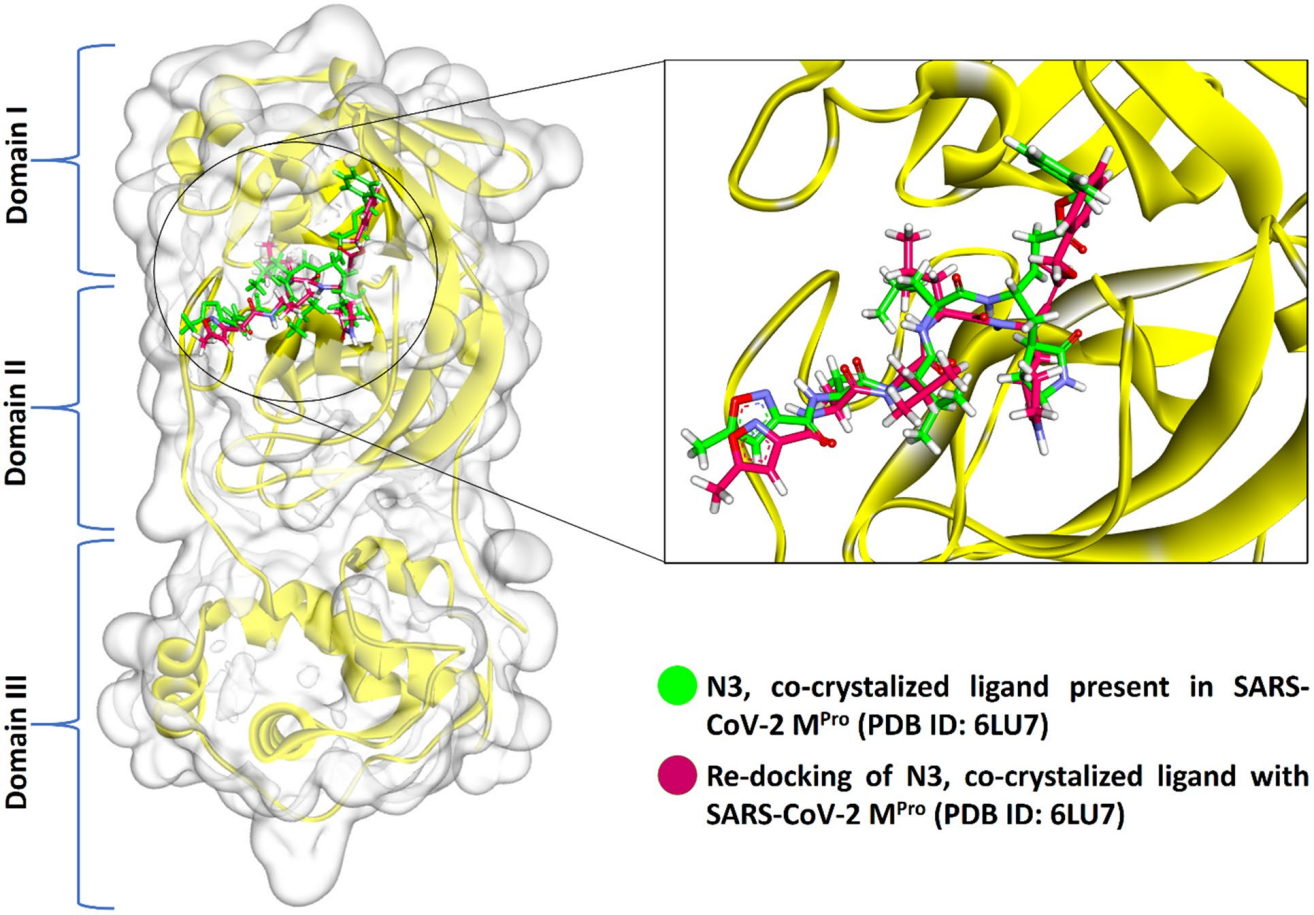
Three-dimensional representation of the co-crystal ligand N3 in the binding cleft of SARS-CoV-2 M^Pro^ (PDB ID: 6LU7). Green color represents the pre-docked pose of co-crystal ligand N3 and re-docked pose of co-crystal ligand N3 was preserved in magenta color (Discovery studio visualizer v21.1.0.20298; https://www.3ds.com/products-services/biovia/).

**Figure 2:**
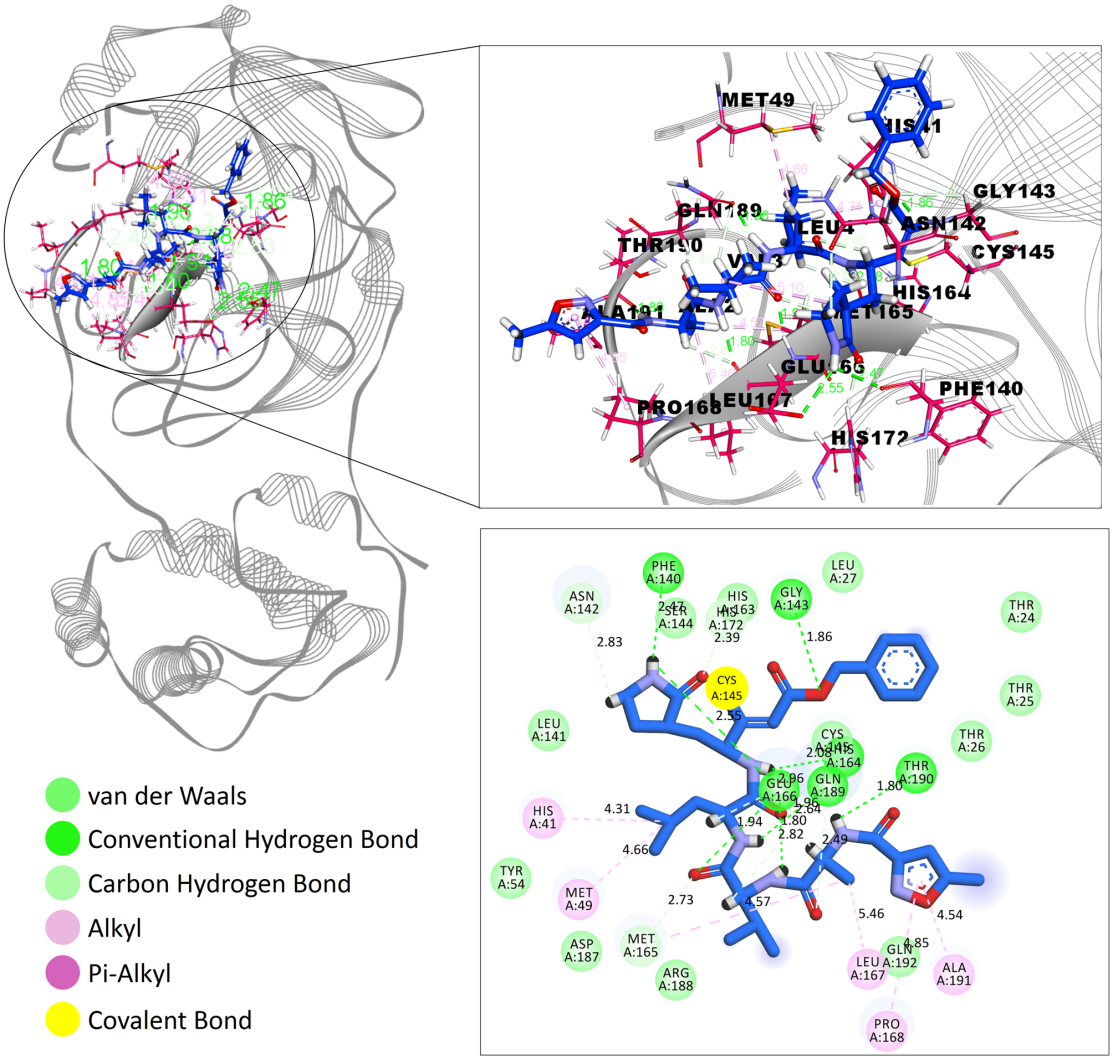
Interaction of N3 in the binding cleft of SARS-CoV-2 M^Pro^ (PDB ID: 6LU7) of COVID-19 shown in (**a**) 3 D representation and (**b**) 2 D representation (for better clarity) describing ligands interactions by formation of various H-bonds and hydrophobic interactions with protein at the active site of the protein (Discovery studio visualizer v21.1.0.20298; https://www.3ds.com/products-services/biovia/).

Virtual screening was performed to identify the best-scoring natural compounds with the ability to inhibit SARS-CoV-2 M^pro^. The N3, the co-crystal ligand of M^pro^ PDB code 6LU7, was redocked to evaluate the ligand binding pose in the catalytic site (Cys-His catalytic dyad) of the M^pro^ enzyme and obtained crystal close pose of N3 confirmed using the RMSD between bound and docked conformations. The M^pro^ enzyme encompasses three domains, domains I and II constitute anti-parallel β-barrel architecture and domain III contain α-helices with an orthogonal bundle-like structure connected to domain II by loop elements [25, I INCLUDED HERE]. Both domains I and II share the N3-binding site of M^pro^ with each protein domain chain contribute half of its cleft to the catalytic pocket. The original position and orientation of N3 in the co-crystallized structure with PDB code 6LU7 and its docked pose is shown in Fig. 1. The best dock pose obtained (binding energy = 7.55 kcal/mol) with an RMSD of 1.70 Å showed the ability of the docking protocol to reproduce native-like ligand poses. N3 is a comparatively large peptide-like molecule that generate various interactions with the amino acid residues of the M^pro^ ligand-binding site viz*.* His164, Met165, Glu166, Leu167, Pro168, Gln189, Thr190 and Ala191 (Fig. 2).

The co-crystallized N3 ligand of the M^pro^ target also embodies a tight covalent bond with Cys145 with 1.8 Å bond length making the inhibitor covalently linked to the protein. The re-docked pose had captured 7 H-bonds (with Gly143, Phe140, His163, His164, Glu166 and Thr190), 1 amide π-stacked (Leu141), 5 π-alkyl (His41, Met49, Leu167, Pro168 and Ala191), 2 carbon-hydrogen bonds (Met165 and His172) and 1 van der Waal (with Asn142) contacts with M^pro^. The generation of native-like ligand poses obtained from N3 peptide re-dock calculations and its binding potential encourages the implementation of docking procedure of 110 lead-like natural compounds within the ligand-binding site of the M^pro^ target. The M^pro^ target contains four sites labelled as S1′, S1, S2, and S4 as part of its ligand-binding site. The inhibitory effect of the M^pro^ enzyme is contributed by the thiol group of the cysteine residue in the S1′ site. Thus, the interaction of Cys145 with the ligand is therefore regarded as an essential component of screening ligand to inhibit the activity of this protease.

**Figure 3:**
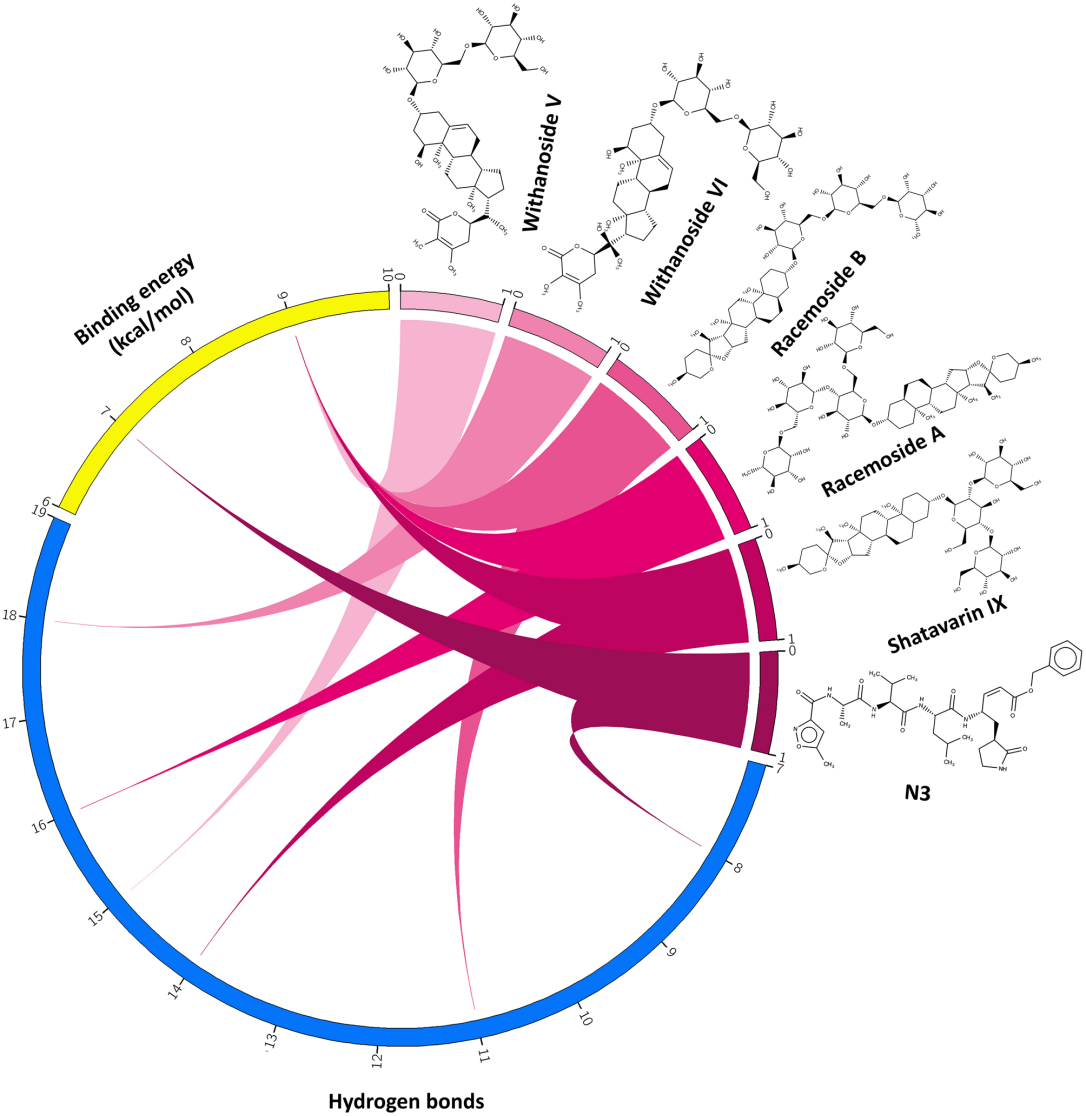
N3, Withanoside-V, Withanoside-VI, Racemoside-B, Racemoside-A, Shatavarin-IX are depicted along with their Hydrogen bonds and Binding energy information using Circos representation. All the three entities—the compounds, Hydrogen bond and Binding energy are shown using three separate ideograms. The six compounds are drawn from light to dark magenta color shades and their respective values of Hydrogen bond (blue color) and Binding energy (yellow color) are displayed using connecting colored ribbons and numbering scales (Circos version 0.69; http://circos.ca/).

**Figure 4:**
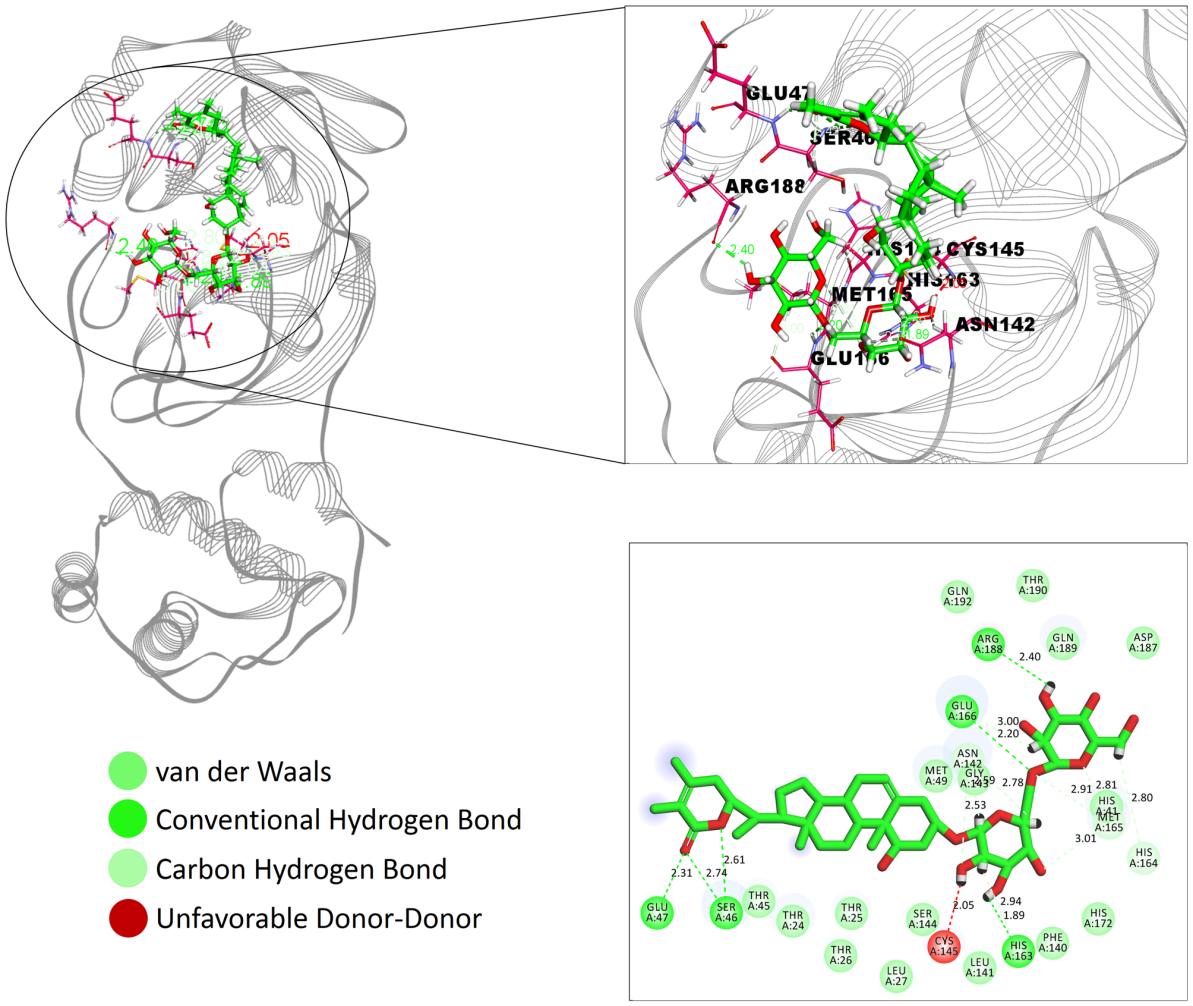
Interaction of Withanoside V in the binding cleft of SARS-CoV-2 M^Pro^ (PDB ID: 6LU7) of COVID-19 shown in (**a**) 3 D representation and (**b**) 2 D representation (for better clarity) describing ligands interactions by formation of various H-bonds and hydrophobic interactions with protein at the active site of the protein (Discovery studio visualizer v21.1.0.20298; https://www.3ds.com/products-services/biovia/).

The natural compounds obtained through docking simulation possessed comparatively better binding affinity as well as key receptor contacts better than co-crystal ligand (N3). We selected an upper threshold for ligand interaction as 8 kcal/mol to select only those phytochemicals better than the co-crystal (7.55 kcal/mol) ligand. Among 110 phytochemicals, top-five compounds were selected, the binding energy of which varied between 10.04 and 8.3 kcal/mol (Fig. 3) and constituted five hydrogen bonds or more with M^pro^ target at the N3 ligand-binding site. These include Withanosides V and VI, Racemosides B and A, and Shatavarin IX with the binding energy values 9.84, 9.74, 9.70, 9.68 and 9.35 kcal/mol, respectively. Several amino acid residues of high interaction frequency that were common among the top five nature molecules were obtained (Thr24, Thr25, Thr26, Leu27, His41, Thr45, Ser46, Glu47, Met49, Phe140, Leu141, Asn142, Gly143, Ser144, Cys145, His163, His164, Met165, Glu166, Leu167, Pro168, His172, Asp187, Arg188, Gln189, Thr190, and Gln192) with a variety of interaction types including hydrogen bonds, hydrophobic contacts, π-stacks, π-alkyl and alkyl contacts (Figs. S1–S4). Withanoside V emerged as the best-scoring inhibitor for SARS-CoV-2 M^pro^ target from virtual screening exercise (Fig. 4).

### In silico ADME/T prediction of top five compounds

The drug likelihood of the top five compounds was assessed in terms of better descriptor scores and key pharmacokinetic properties calculated using the Schrodinger QikProp module. N3 was also subjected to QikProp ADME/T profiling and select ADME/T properties are given in Table 1. The top five compounds displayed drug-like traits based on physico-chemical properties. The ADME/T prediction studies revealed that the top five compounds comply with the Lipinski rule of five. They constitute the comparable log P values for the drug absorption and no violation was observed in the default range of physicochemical and ADME/T parameters. Certain properties such as blood–brain permeability and percentage of human oral absorption were also computed within the acceptable limits as specified for Human usage. Intriguingly, this analysis also showed that Withanoside-V possesses the largest polar surface area (301.363 Å^3^) within the top five docked compounds suggesting the best compound among the top five docked compounds with considerable structural similarity.

**Table 1:** QikProp calculation of physico-chemical properties and ADME/T properties.

Compounds	Rule of five	PSA	QPlogPo/w	QPpolrz	QPlogBB	QPlogKp
Withanoside-V	No violation	301.363	− 2.036	86.641	− 4.397	− 5.831
Withanoside-VI	No violation	246.197	0.004	80.703	− 4.735	− 6.273
Racemoside-B	No violation	259.608	− 0.992	77.748	− 4.722	− 6.38
Racemoside-A	No violation	241.348	− 1.014	67.872	− 4.843	− 6.817
Shatavarin-IX	No violation	224.561	− 0.526	67.865	− 4.757	− 6.397

*PSA* Van der Waals surface area of polar atoms (7 < PSA < 200), *QP log Po/w* predicted octanol/water partition coefficient (− 2 QPlogPo/w 6.5), *QPpolrz* predicted polarizability (13.0–70.0), *QP log BB* predicted brain/blood partition coefficient (− 3 < QPlogBB < 1.2), *QP log Kp* predicted skin permeability (− 8.0 to − 1.0).

### Molecular dynamics simulation

**Figure 5:**
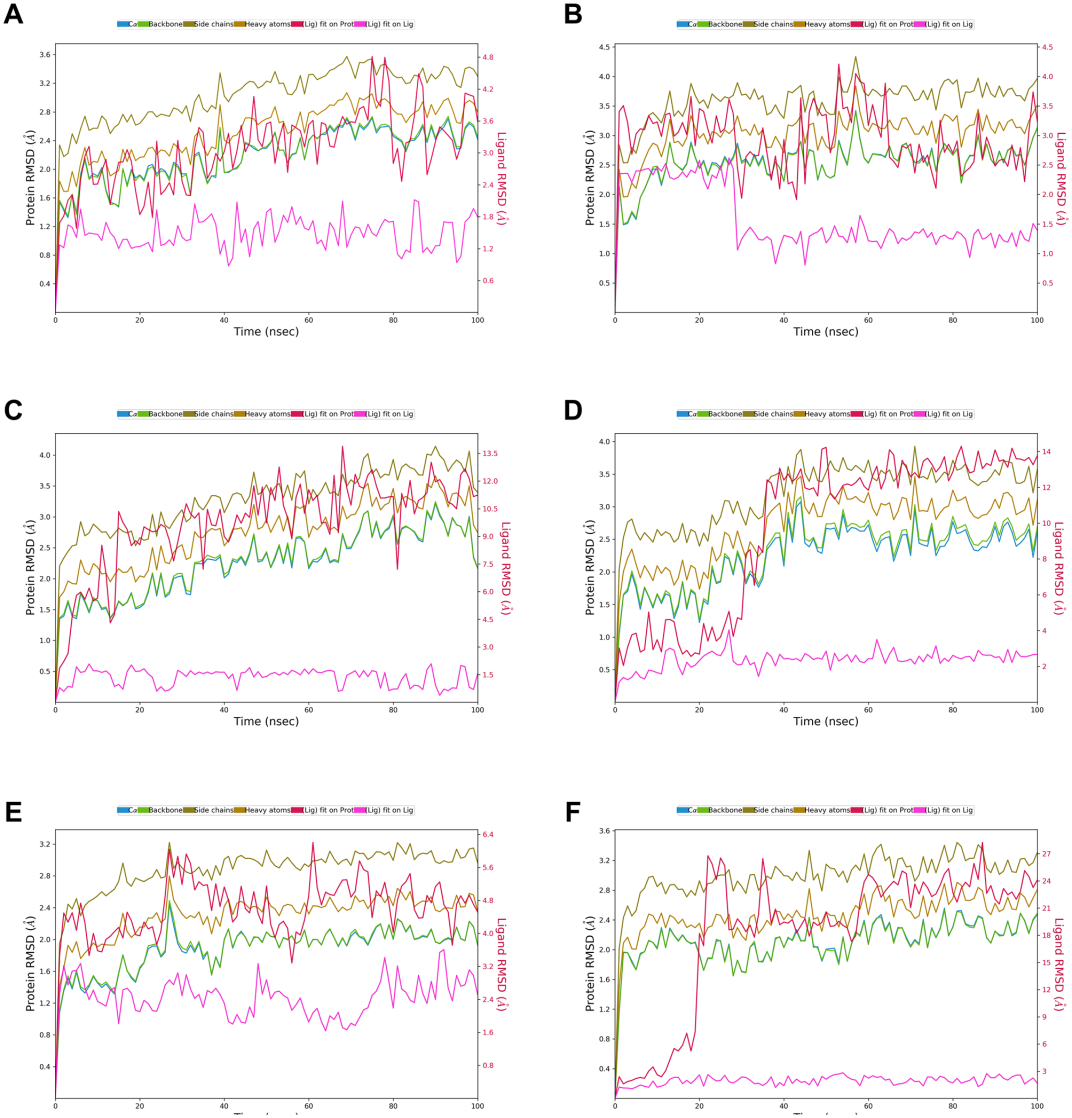
RMSD plot of SARS-CoV-2 M^Pro^ (PDB ID: 6LU7) of COVID-19 target with co-crystal ligand and top-five phytochemicals as a function of simulation time. (**A**) N3, (**B**) Withanoside-V, (**C**) Withanoside-VI, (**D**) Racemoside-B, (**E**) Racemoside-A, (**F**) Shatavarin-IX (Desmond Molecular Dynamics System, D. E. Shaw Research v6.1; https://www.deshawresearch.com/resources_desmond.html/).

**Figure 6:**
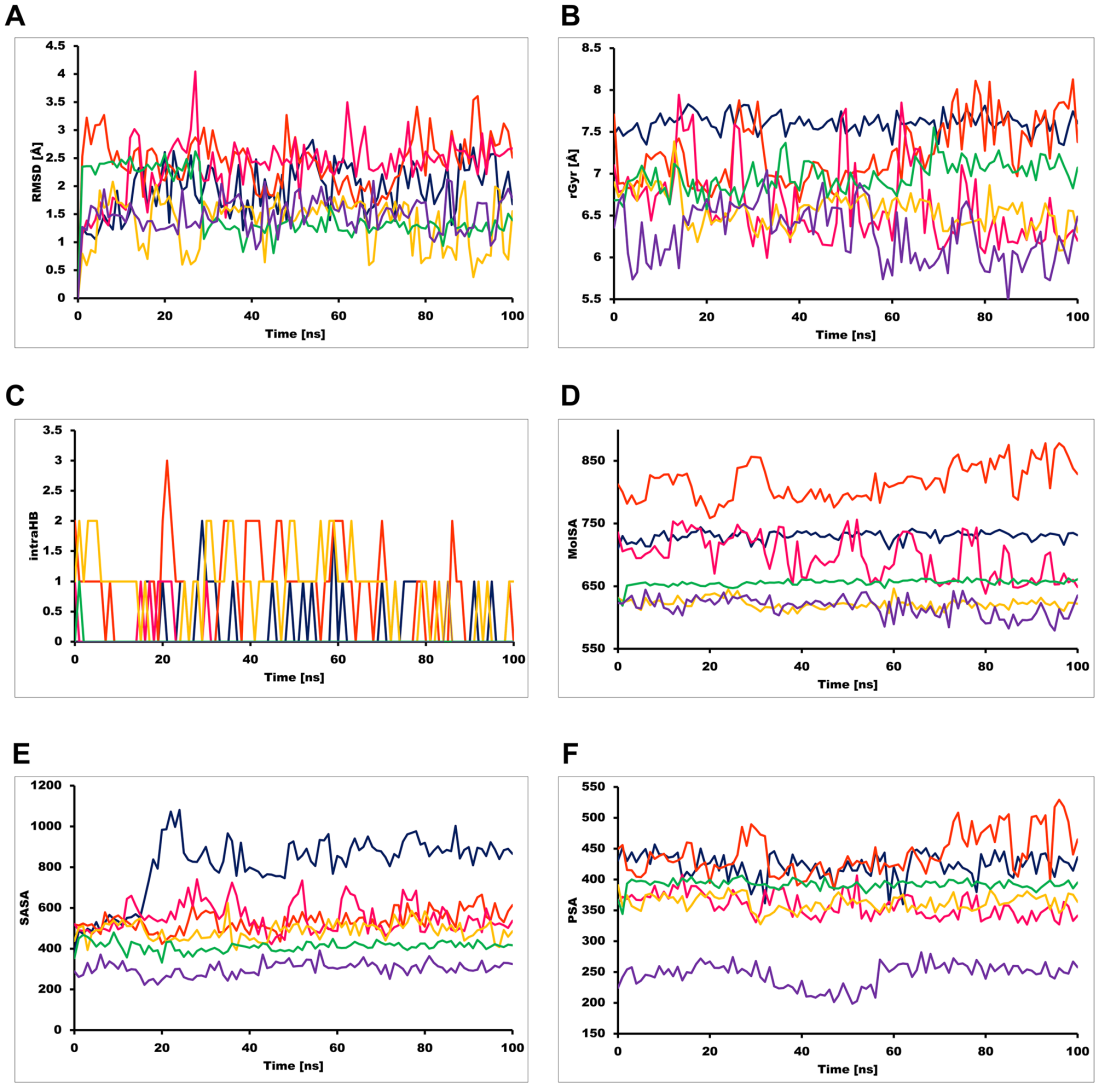
Various measures of the molecular dynamics simulations of co-crystal ligand and top-five phytochemicals with SARS-CoV-2 M^Pro^ target. (**A**) RMSD, (**B**) rGyr, (**C**) intra HB, (**D**) MolSA, (**E**) SASA and (**F**) PSA. N3 (purple color), Withanoside-V (green color), Withanoside-VI (yellow color), Racemoside-B (magenta color), Racemoside-A (orange color), Shatavarin-IX (blue color) (Desmond Molecular Dynamics System, D. E. Shaw Research v6.1; https://www.deshawresearch.com/resources_desmond.html/).

The best-docked complexes were obtained from the virtual screening experiment to investigate the conformational stability and time-dependent binding ability of ligands in the M^pro^ catalytic pocket. Although virtual screening generates the spatial orientation of the ligand within the receptor pocket roughly, further accounting factors such as binding affinity and conformational stability needs to be evaluated rigorously to assess the inhibitory potency of natural compounds targeting M^pro^ in addition to the binding pocket fitness. Conformational stability of ligand is essential for ligand-mediated inhibition of M^pro^ and analysis of molecular dynamic (MD) simulations offers a thorough study of conformational landscape of protein–ligand complexes close to real physiological condition. Independent MD simulations were carried out for M^pro^ target protein in complex with N3, Withanosides V and VI, Racemosides A and B and Shatavarin IX using the Schrodinger (v. 2020-4) Desmond module. The MD trajectory obtained was used to investigate the equilibrium of dynamics over a function of time. An insight into the convergence of simulated protein–ligand complexes can be achieved by taking into account the root mean square deviations (RMSD) metric of the initial structure and the average simulated structure of all MD trajectory frames. Protein–ligand RMSD plots for all top scoring molecules demonstrated the stability of the docked complexes which achieved only after 17 ns which is in good agreement with the simulation of N3 co-crystal ligand. Further, RMSD variations were ~ 3 Å for all-natural compounds (Fig. 5). Compared to RMSD plots, RMSF protein fluctuations were highest in the residue index window at 30–50, 150–160, 260–280, 190–310 positions as some of the amino acid residues of this window were pocket residues promoting ligand binding (Figs. S5–S10). The secondary structural elements corresponding to pocket residues were intact in the β-sheets and loop regions of M^pro^ (Figs. S11–S16). A thorough examination of RMSD of these fluctuating residues was found to be enriched with loop structure that showed up peaks about ~ 3 Å in respective plots. The ligand RMSD plots revealed fluctuation for all compounds were in the range between 0.5 and 4 Å (Fig. 6A). The compactness calculated by the radius of gyration parameter showed similar notions for all compounds excluding Withanoside V and Shatavarin IX (Fig. 6B). Intramolecular hydrogen bonding was detected in the compounds Withanoside VI, Racemoside A and Shatavarin IX (Fig. 6C) which accounted for stronger contacts for prolonged binding residence within the ligand-binding site. The molecule surface area (MSA) of Withanoside V was around 650 Å^2^ in most of the frames in the trajectory (Fig. 6D). The solvent-accessible surface area (SASA) and polar surface area (PSA) for Racemoside B and Shatavarin IX were more than 600 Å^2^ in most of the intervals (Fig. 6E,F). Both compounds were better fitted to the M^pro^ target pocket while the co-crystal ligand N3 did not such establish strong contacts during simulation; N3 PSA did not lead to hydrogen bonding and was largely exposed to solvents in most of the trajectory frames.

### Preservation of intermolecular contacts in molecular dynamics simulations

**Figure 7:**
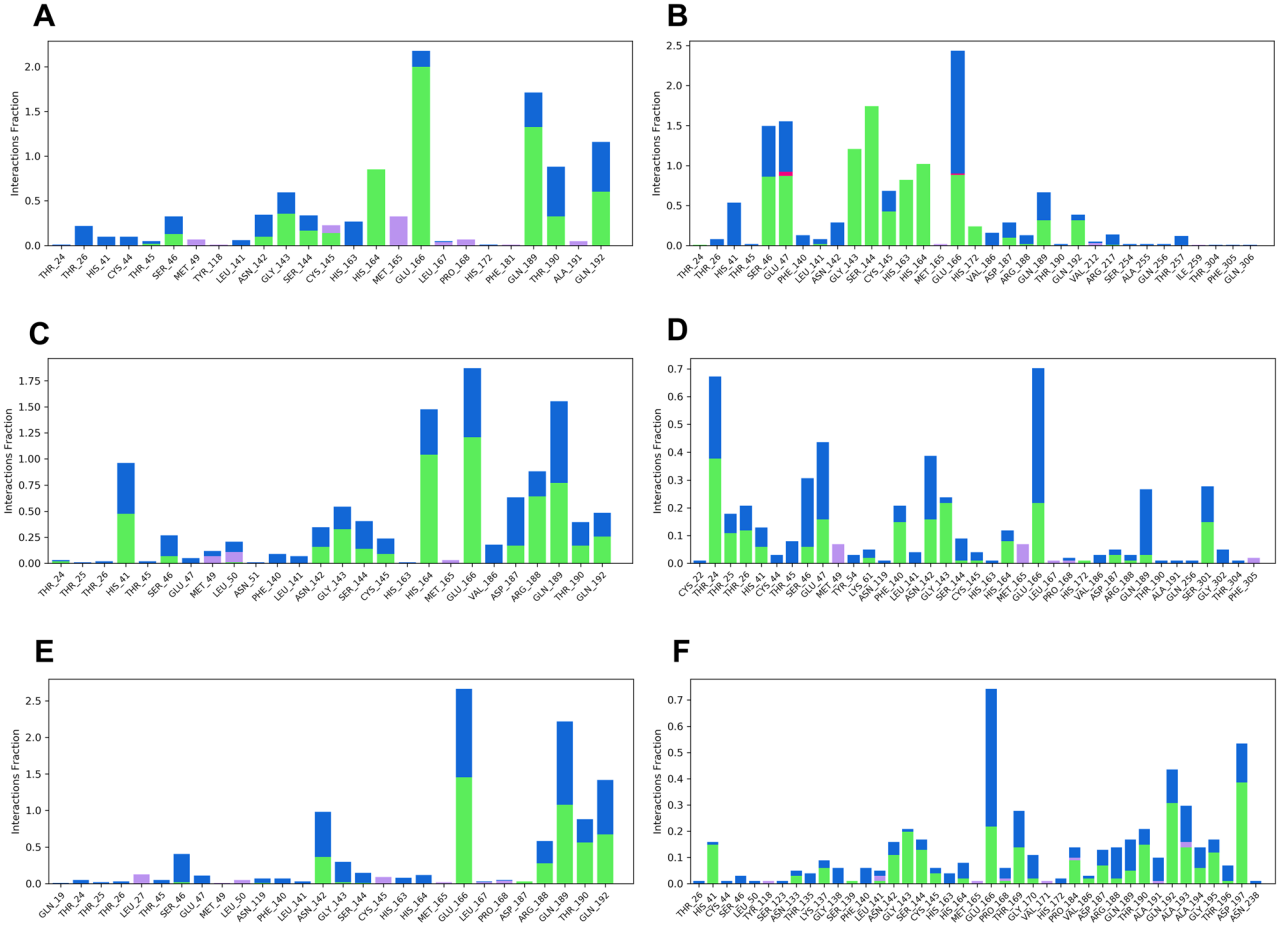
Various intermolecular interactions made by SARS-CoV-2 M^Pro^ pocket residues with co-crystal ligand and top-five phytochemicals compounds, captured during molecular dynamics simulations. (**A**) N3, (**B**) Withanoside-V, (**C**) Withanoside-VI, (**D**) Racemoside-B, (**E**) Racemoside-A, (**F**) Shatavarin-IX (Desmond Molecular Dynamics System, D. E. Shaw Research v6.1; https://www.deshawresearch.com/resources_desmond.html/).

**Figure 8:**
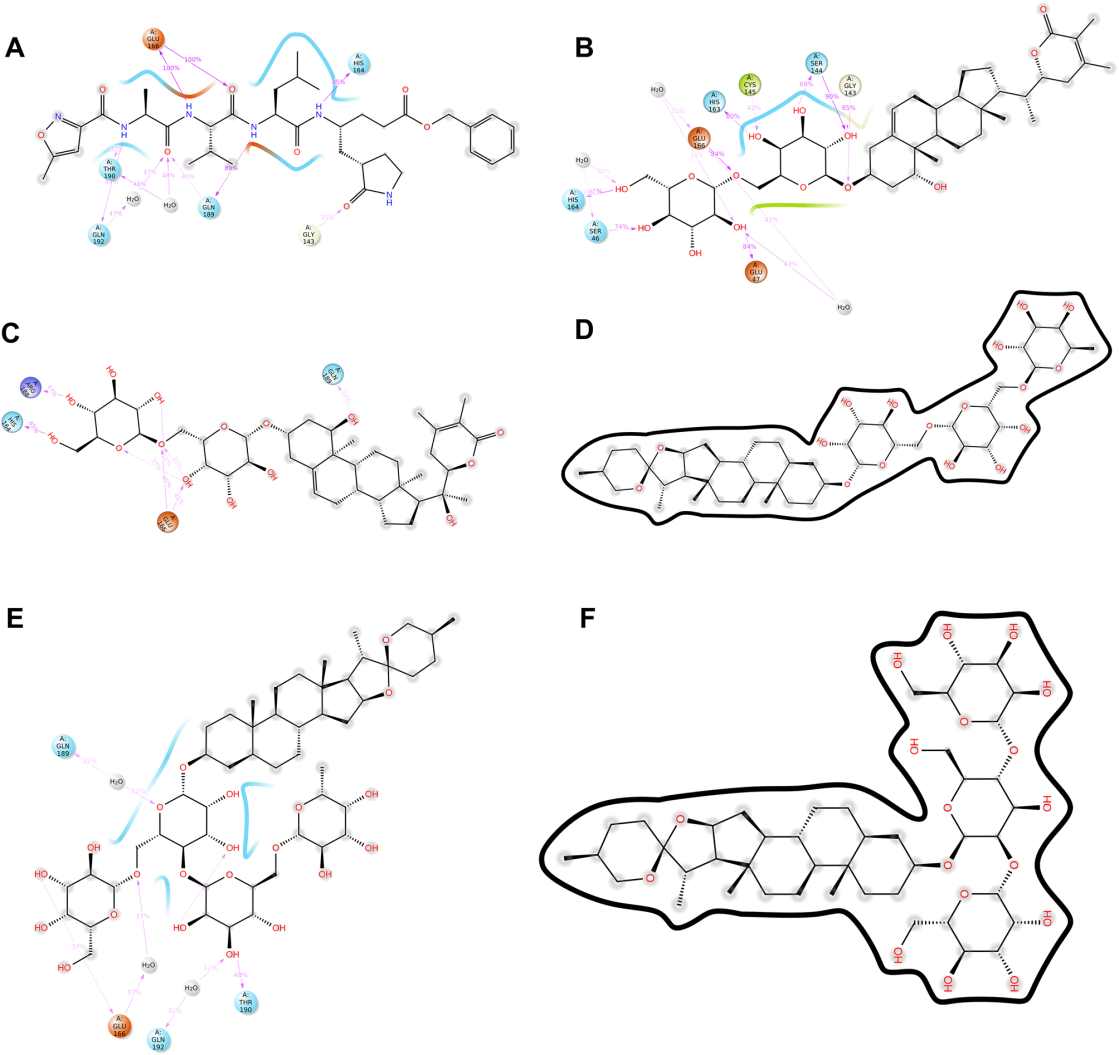
Preserved contacts of co-crystal ligand and top-five phytochemicals with SARS-CoV-2 M^Pro^ target, captured during molecular dynamics simulations (Desmond Molecular Dynamics System, D. E. Shaw Research v6.1; https://www.deshawresearch.com/resources_desmond.html/).

The crystal structure of SARS-CoV-2 M^pro^ with N3 indicated that its ligand-binding site consists of an adjacent hydrophobic sub-pocket lined by Met49, Met165, Leu167, Pro168 and Ala191 residues. N3 also developed hydrogen bonds with 11 amino acids viz*.* Thr45, Ser46, Asn142, Gly143, Ser144, Cys145, His164, Glu166, Gln189, Thr190 and Gln 192. The N3-mediated water bridges were also noticed with residues in the windows of Thr24 to His163 and Gln189 to Gln192. Figure 7 illustrates the various types of intermolecular interactions developed by N3 peptide including hydrogen bond, hydrophobic and water bridges. The 2D interaction charts of re-docked and top five natural compounds depicting the retention of contacts along the simulation course are plotted in Fig. 8. N3 preserved almost the entire set of crystal contacts viz*.* Glu166 and Gln189 alkyl moiety (negative charge, 100% and Polar charge, 89%), His164 (water-bridges, 56%), Gly143 (carboxylate group, 35%). Indeed, all these intermolecular interactions which were captured during the simulations improved the robustness of the docking procedure in generating a stable dock pose (Fig. 2). Withanoside V was identified as the best-scoring compound which developed 1 hydrophobic contact with Cys145 (42%), cyclopenta phenathrene pyran ring with polar (Ser144, 90%, His163, 80%, Ser46, 74%) and negatively charged amino acids (Glu47, 84% and Glu166, 84%) (Fig. 8). Withanoside V possessed the same cyclopenta phenanthrene pyran moiety with positively charged (Arg188, 47%), negatively charged (Glu166, 45%) and 1 polar contact with His164, 65%, the highest profile of preserved contacts comparatively (Fig. 8C).

Racemoside B in its docking profile developed five hydrogen contacts (Thr26, Cys145, His163, His164 and Glu166) and four alkyl interactions with Met165, Pro168 and Ala191 which were observed in the intermolecular interaction profiles. Based on this observations, one unfavorable bond was identified with Thr24 residue and maximum interactions were made at Thr24 to Thr304 residue window (Fig. 7D). It also developed alkyl contacts with Met165, Pro168 and Ala191 with one π-alkyl with Pro168. Racemoside A developed eleven hydrogen bonds (one with Phe140, Leu141, Ser144, Cys145, His163, Gln192, Thr190 and four with Glu 166 during the docking experiment. The tetramethyl-oxapino (oxane) [pentacyclo] icosane generated one alkyl and one π-alkyl contact with His41 and Met49, respectively. One polar and negatively charged contact were also developed which retained 37% of docking pose interactions (Fig. 8E). Shatavarin IX had produced seven hydrogen bonds, one alkyl and one unfavorable donor–donor interaction in its docking pose which was not retained in the MD simulation event.

### Binding free energy calculations of selected natural compounds with SARS-CoV-2 M^pro^ target

**Figure 9:**
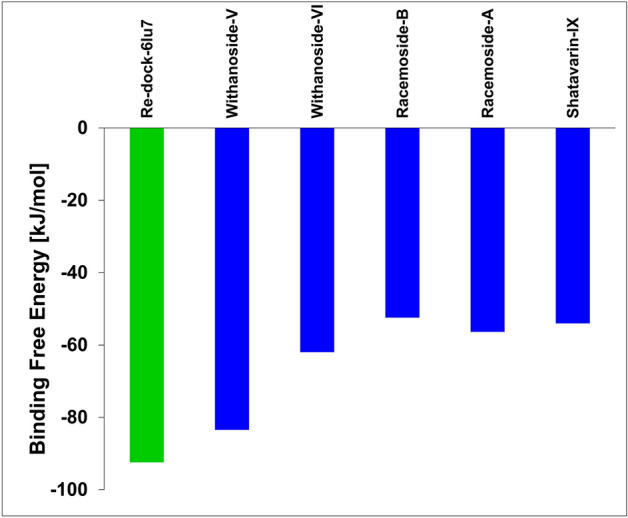
Binding free energy calculations of co-crystal ligand and top-five phytochemicals compounds with SARS-CoV-2 M^Pro^ target. The co-crystal ligand of SARS-CoV-2 M^Pro^ target, N3, is shown in green color bar. The phytochemicals are represented in blue color bars (Microsoft 365 Excel v2102; https://www.microsoft.com/en-us/microsoft-365).

The free energy of binding of top five natural compounds with SARS-CoV-2 M^pro^ was calculated using the MM/PBSA approach. The simulation trajectory of 100 ns time scale was supplied to the YASARA binding energy macro. The cognate peptide N3 secured − 92.43 kJ/mol whereas Withanoside V obtained the binding energy of − 83.45 kJ/mol (Fig. 9). This observation highlighted that N3 developed better crystal contacts along with new contacts resulting in securing the top rank in the energy list among the selected natural compounds. It created new hydrogen bonds with Gln 192 residue which can be beneficial. Withanoside V ranked in second place which developed one hydrophobic interaction with Cys145 and one hydrogen bond with Gly143. Withanoside VI ranked in the third position with the binding free energy of − 61.97 kJ/mol while Racemoside B, Racemoside A and Shatavarin IX scored − 52.44, − 56.35 and − 53.99 kJ/mol, respectively.

## Materials and methods

### Dataset preparation

A library of 110 natural compounds with experimental antiviral properties were manually compiled from literature with special emphasis on plants including *Withania somnifera, Asparagus racemosus, Zinziber officinalis, Allium sativum, Curcuma longa,* and *Adhatoda vasica*^39–41^*.* The 3D structure of these natural compounds was retrieved from the NCBI PubChem database or by drawing the structure using a MarvinSketch 20.20.1 (ChemAxon, LLC) drawing tool if unavailable. The ligand library was prepared for docking by atom-typing, removing crystallographic water molecules and by adding explicit hydrogen and assigning charges. These structures were then energy-minimized using the steepest gradient approach (100 iterations) and docked in the ligand-binding site using YASARA Structure (academic license 20.4.24) with Amber03 force field. The crystal structure of SARS-CoV-2 main protease (M^pro^) was retrieved from Protein Databank (PDB code: 6LU7, 2.16 Å resolution). Single chain of M^pro^ with bound N3 peptide was chosen as the structure for docking calculations and dynamic simulations^42^.

### Virtual library screening upon SARS-CoV-2 M^pro^ target

Virtual screening was carried out to screen the prepared library of 110 natural compounds towards N3-binding site using YASARA Structure (academic license 20.4.24). YASARA Structure utilizes AutoDock Vina^43^ as the pose searching technique and AMBER03 force field to score the resulting dock poses. Re-docking of the N3 co-crystal ligand was performed to verify whether Vina generates native-like dock poses. A simulation cell enclosing the N3-binding site was created (dimensions: 20 × 20 × 20 Å) to generate receptor grid generation suitable for Vina docking. The dock results were ranked on the basis of docking score from high to low to identify the top-scoring natural compounds^44^. The YASARA empirical energy function computes the intermolecular interactions between a receptor and a ligand by calculating the difference between the sum of potential energy and solvation energy terms in free state (receptor and ligand in isolation) and the sum of same set of energy terms in the complex state (ligand bound receptor conformation) using AMBER3 force field leading to binding affinity with positive sign^45^. Therefore, binding energy with more positive energy indicates higher affinity of ligands bound to receptors^46^. The binding energy (Δ*G*_bind_) of protein–ligand interactions was calculated using the following empirical equation:

1 $$\Delta G = \Delta G_{vdW} + \Delta G_{Hbond} + \Delta G_{elec} + \Delta G_{tor} + \Delta G_{desolv}$$

where Δ*GvdW* is the van der Waals term for docking energy; Δ*GHbond* is the H bonding term fordocking energy; Δ*Gelec* is the electrostatic term for docking energy; Δ*Gtor* is the torsional free energy term for ligand when the ligand transits from unbounded to bounded state; Δ*Gdesolv* is the desolvation term for docking energy.

### In silico physico-chemical and ADME/T studies

Physico-chemical and ADME/T properties were calculated using the Qikprop module of Schrodinger suite 2020-4. The physico-chemical and ADME/T properties of the top five best-scoring compounds were computed to understand its pharmacokinetic role^47^.

### Molecular dynamic simulations of the top-scoring molecule with M^pro^ target

The molecular dynamic (MD) simulation of M^pro^-ligand complexes was performed using the Desmond module of Schrödinger (release 2019-3, academic version). The MD simulations of re-docked (N3) and top-scoring natural compounds were carried out for 100 ns time interval each. Initially, the receptor–ligand complexes were prepared using the Protein Preparation Wizard of Schrödinger suite with default settings including reassignment of hydrogens, and bonds, addition of missing side chain atoms of amino acid residues, and optimization of loops residues, and sampling of water orientation at pH 7.4. A periodic simulation box was created using the System Builder module, solvated the system using TIP3P (Transferable Intermolecular Potential with 3 Points) water model and neutralized by adding counter ions, energy minimization with 1000 iterations of the steepest descent technique using the OPLS (Optimized Potentials for Liquid Simulations) all-atom force field. After equilibrium, an unrestrained production phase with NPT (atoms, pressure and temperature were kept constant) ensemble was progressed for 100 ns monitored by Nosé–Hoover thermostat (300 K, relaxation time = 1 ps) and isotropic Martyna–Tobias–Klein barostat (1.01325 bar, relaxation time = 2 ps). Short-range interactions (cut-off = 9 Å) and long-range Coulomb interaction were evaluated using the smooth particle mesh Ewald (PME) method with the RESPA integrator. The frames capturing the dynamic motions of the system were exported at the interval of 5 ps. The stability of the system was studied by plotting histograms for RMSD, root mean square fluctuations (RMSF), Hydrogen bond analysis, radius of gyration (Rg) and torsional bonds^45,48^.

### Binding free energy calculations of top-scoring molecules with SARS-CoV-2 M^pro^ target

The single trajectory method was used to compute free energy of binding using MM/PBSA (Molecular Mechanics/Poisson-Boltzmann Surface Area) method with binding energy macro of YASARA Structure. Adaptive Poisson–Boltzmann Solver with AMBER14 APBS force field was employed to calculate electrostatics and solvation energies of the simulation objects^49,50^. The 100 ns long simulation trajectory of the top five natural compounds and the co-crystal ligand was supplied as the input to macro.

2 $$\Delta G_{bind} = \Delta G_{complex(minimized)} {-}\left[ {\Delta G_{ligand(minimized)} + \Delta G_{receptor(minimized)} } \right]$$

and

3 $$\Delta G_{bind} = \Delta G_{MM} + \Delta G_{PB} + \Delta G_{SA - T\Delta S}$$

where Δ_*TDS*_ is the conformation entropic contribution, and Δ*G*_*MM*_ is the molecular mechanics' interaction energy (electrostatic + van der Waals interaction) between protein and ligand. Δ*G*_*PB*_ and Δ*G*_*SA*_ indicate the polar solvation energy and the nonpolar solvation energy, respectively.

## Conclusion

The unavailability of a more effective drug has already exacerbated the condition of COVID-19 pandemic. Several efforts are being made to target the first step of viral invasion and infection of SARS-CoV-2 enabled by the molecular interactions between human Angiotensin-converting enzyme 2 and CoV-2 spike proteins. The rapidly evolving mechanism of CoV-2 brought out by different variants direct our focus to target essential viral enzymes for multiplication. M^pro^ is one such vital enzymes and forms as a viable strategy for developing inhibitors assisted by computational search for inhibitors with promising ADME/T profiles. This study prioritizes select lead compounds against the SARS-CoV-2 M^pro^ from a compiled natural compound library using a hierarchical protocol for molecular docking, dynamics simulations and binding free energy calculations. Withanoside V and VI, Racemoside A and B, and Shatavarin IX have emerged as the robust natural compounds with a stronger binding affinity profile. Dynamic simulations of docked complexes for 100 ns time scale highlighted the structural integrity of protein–ligand complexes. Various parameters of simulations have jointly assisted in elucidating the better binding of natural compounds with the M^pro^ target. Certain top-ranked compounds also retained M^pro^ crucial interactions similar to N3 cognate peptide. Further, the MM/PBSA calculations demonstrated that contribution of binding free energy to individual amino acids residues of the M^pro^ pocket is predominantly governed by hydrophobic residues. This report offers valuable perspectives at the preliminary stage of the SARS-CoV-2 M^pro^ drug design; the hierarchical approach used in this study can be used to identify and refine natural molecules which need further experimental confirmation via in vitro and in vivo studies.

## Supplementary Information

Supplementary Information.
