# Supplementary material for: Identification of antiviral phytochemicals as a potential SARS-CoV-2 main protease (Mpro) inhibitor using docking and molecular dynamics simulations
Source: Sci Rep. 2021 Oct 13;11:20295. doi: 10.1038/s41598-021-99165-4 (PMC8514552; doi:10.1038/s41598-021-99165-4)
Supplement: Supplementary file 1 — Supplementary Information. [file 41598_2021_99165_MOESM1_ESM.docx]

**Identification of antiviral phytochemicals as a potential SARS-CoV-2 Main protease (M^pro^) inhibitors using docking and molecular dynamics simulations**

**Chirag N. Patel^1^, Siddhi P. Jani^1^, Dharmesh G. Jaiswal^1^, Sivakumar Prasanth Kumar^1^, Naman Mangukia^1,2^, Robin M. Parmar^3^, Rakesh M. Rawal^4^ and Himanshu A. Pandya^1^***

^1^Department of Botany, Bioinformatics, and Climate Change Impacts Management, School of Sciences, Gujarat University, Ahmedabad-380009, India.

^2^BioInnovations, Bhayander (West), Mumbai-401101, India

^3^Department of Zoology, School of Sciences, Gujarat University, Ahmedabad-380009, India.

^4^Department of Life Science, School of Sciences, Gujarat University, Ahmedabad-380009, India.

**Details of correspondence**

Prof. (Dr.) Himanshu A. Pandya

Department of Botany, Bioinformatics, and Climate Change Impacts Management, School of Sciences, Gujarat University, Ahmedabad 380009, Gujarat, India.

Email: [hapandya@gujaratuniversity.ac.in](mailto:hapandya@gujaratuniversity.ac.in)

**Supplemental Files**

**Figures**


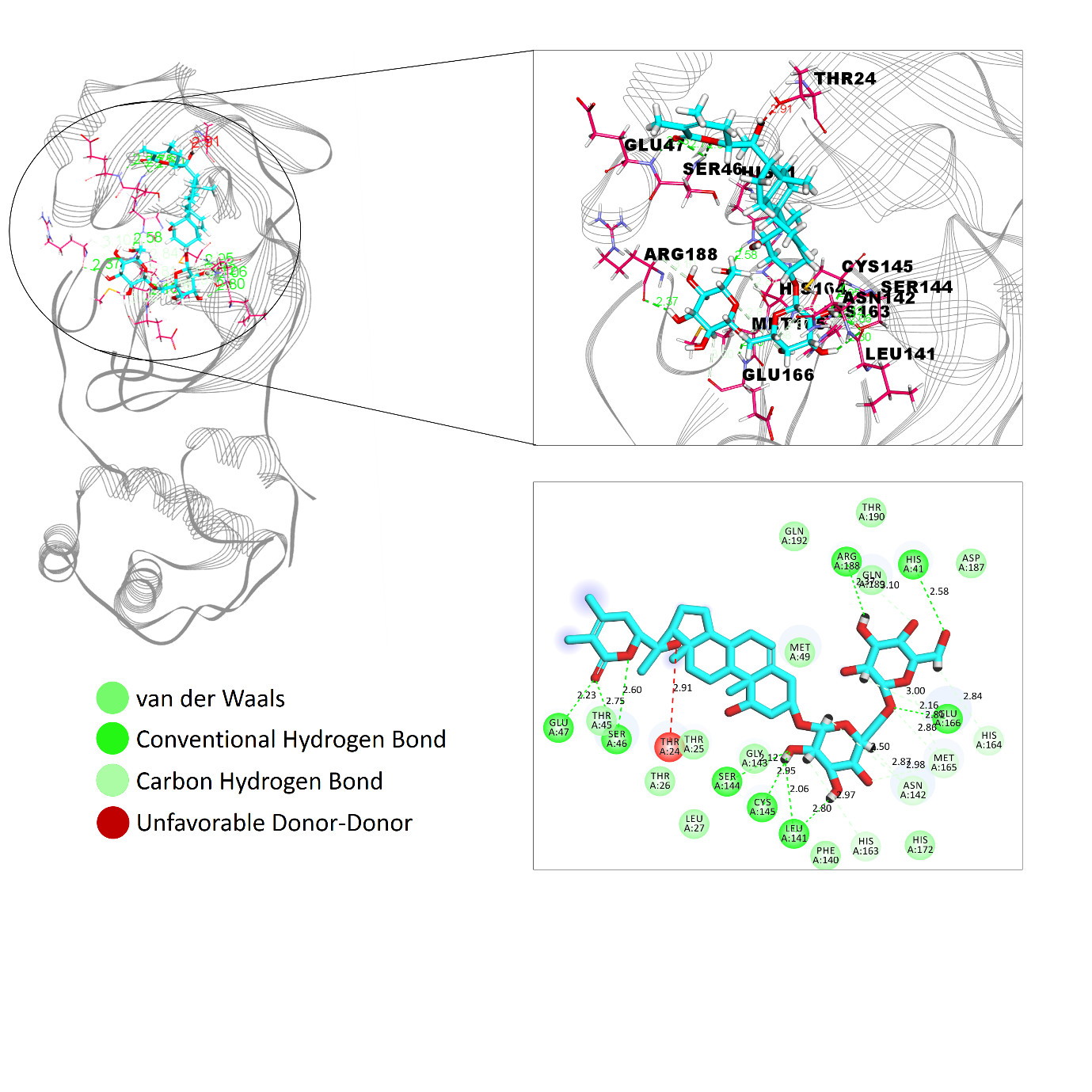


**Fig-S1.** Interaction of Withanoside VI in the binding cleft of SARS-CoV-2 M^Pro^ (PDB ID: 6LU7) of COVID-19 shown in (a) 3 D representation and (b) 2 D representation (for better clarity) describing ligands interactions by formation of various H-bonds and hydrophobic interactions with protein at the active site of the protein.


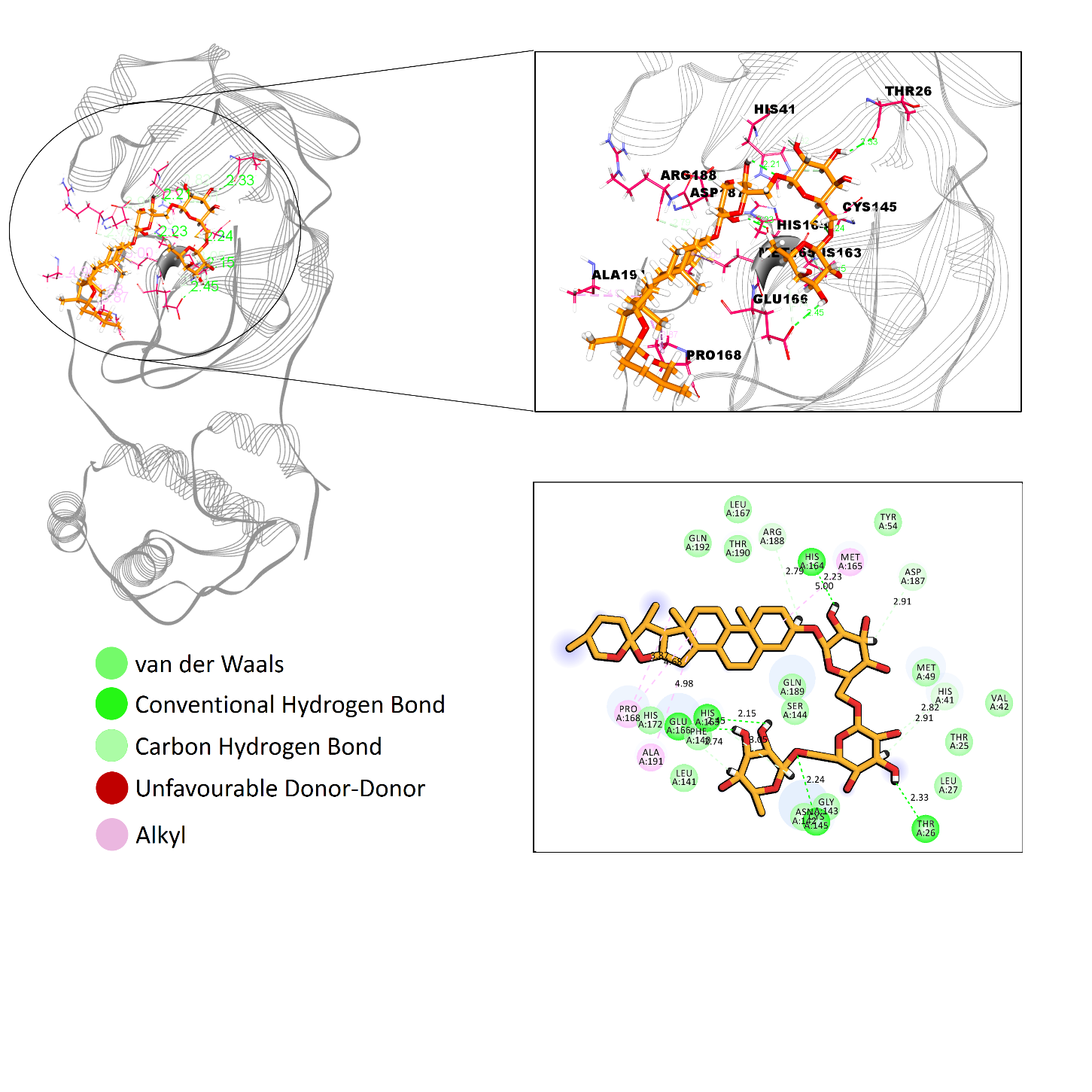


**Fig-S2.** Interaction of Racemoside B in the binding cleft of SARS-CoV-2 M^Pro^ (PDB ID: 6LU7) of COVID-19 shown in (a) 3 D representation and (b) 2 D representation (for better clarity) describing ligands interactions by formation of various H-bonds and hydrophobic interactions with protein at the active site of the protein.


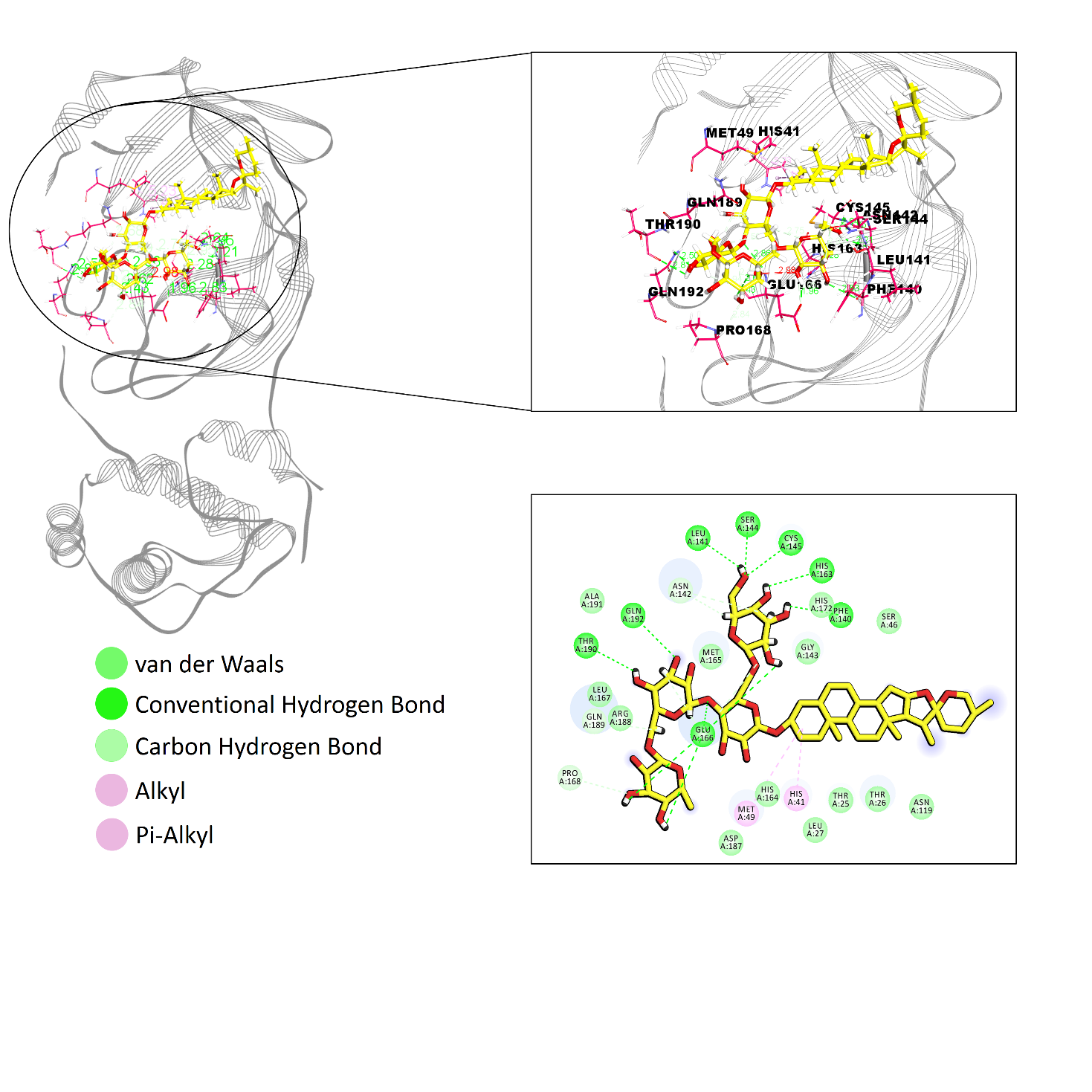


**Fig-S3.** Interaction of Racemoside A in the binding cleft of SARS-CoV-2 M^Pro^ (PDB ID: 6LU7) of COVID-19 shown in (a) 3 D representation and (b) 2 D representation (for better clarity) describing ligands interactions by formation of various H-bonds and hydrophobic interactions with protein at the active site of the protein.


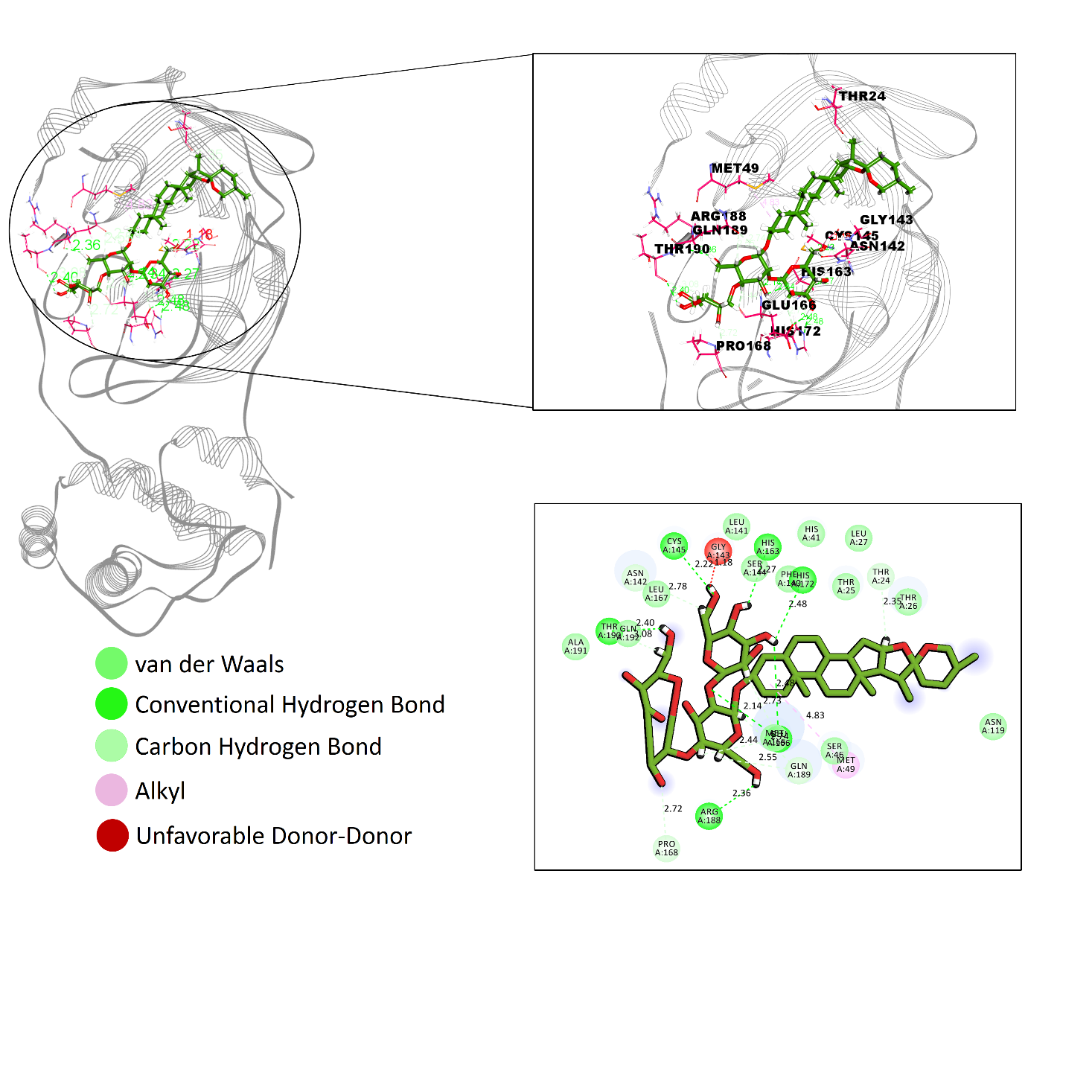


**Fig-S4.** Interaction of Shatavarin IX in the binding cleft of SARS-CoV-2 M^Pro^ (PDB ID: 6LU7) of COVID-19 shown in (a) 3 D representation and (b) 2 D representation (for better clarity) describing ligands interactions by formation of various H-bonds and hydrophobic interactions with protein at the active site of the protein.


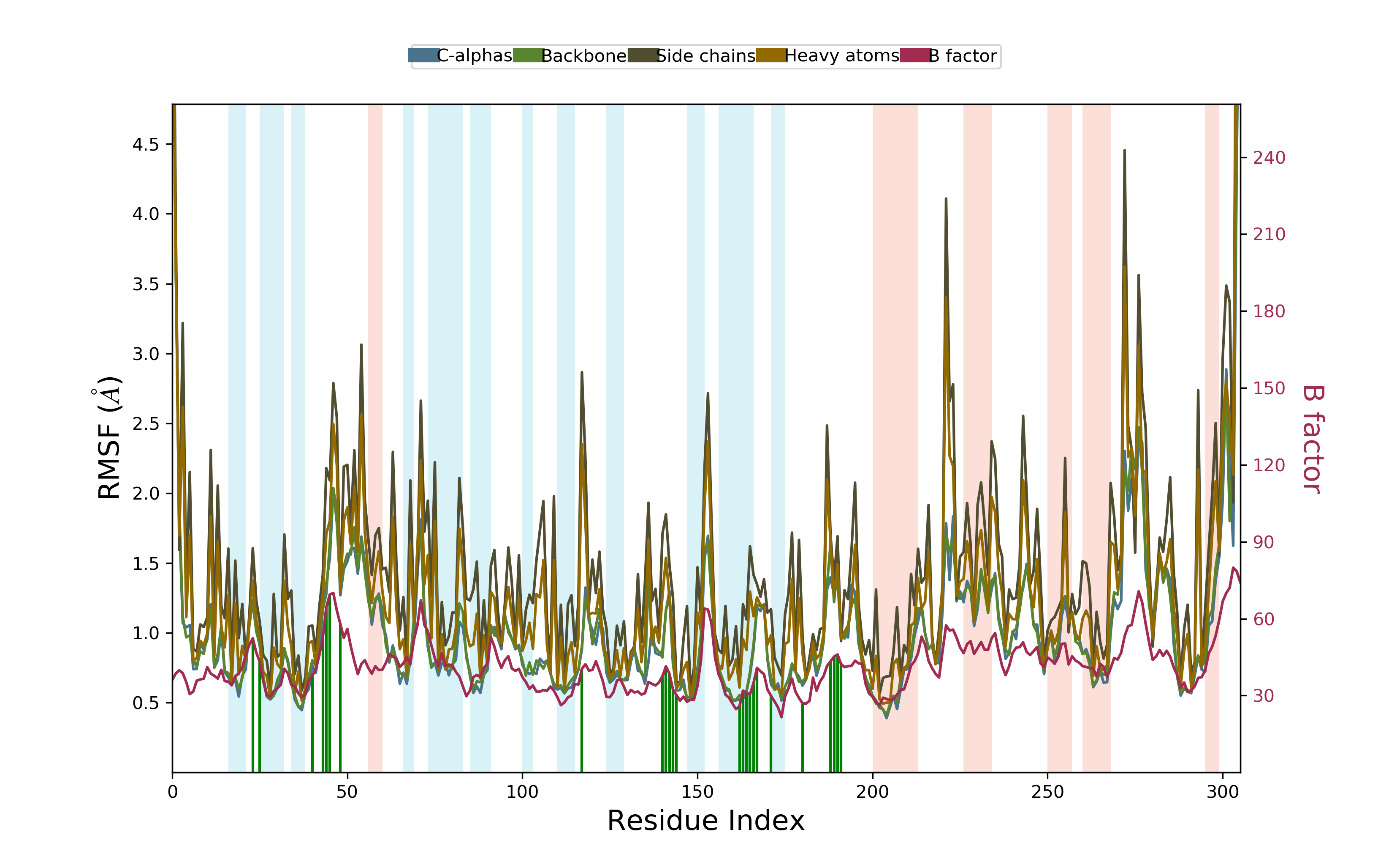


**Fig-S5.** RMSF plot of SARS-CoV-2 M^Pro^ target in complex with N3. The green bar plotted in the protein residue index corresponds to highly fluctuating amino acid residues.


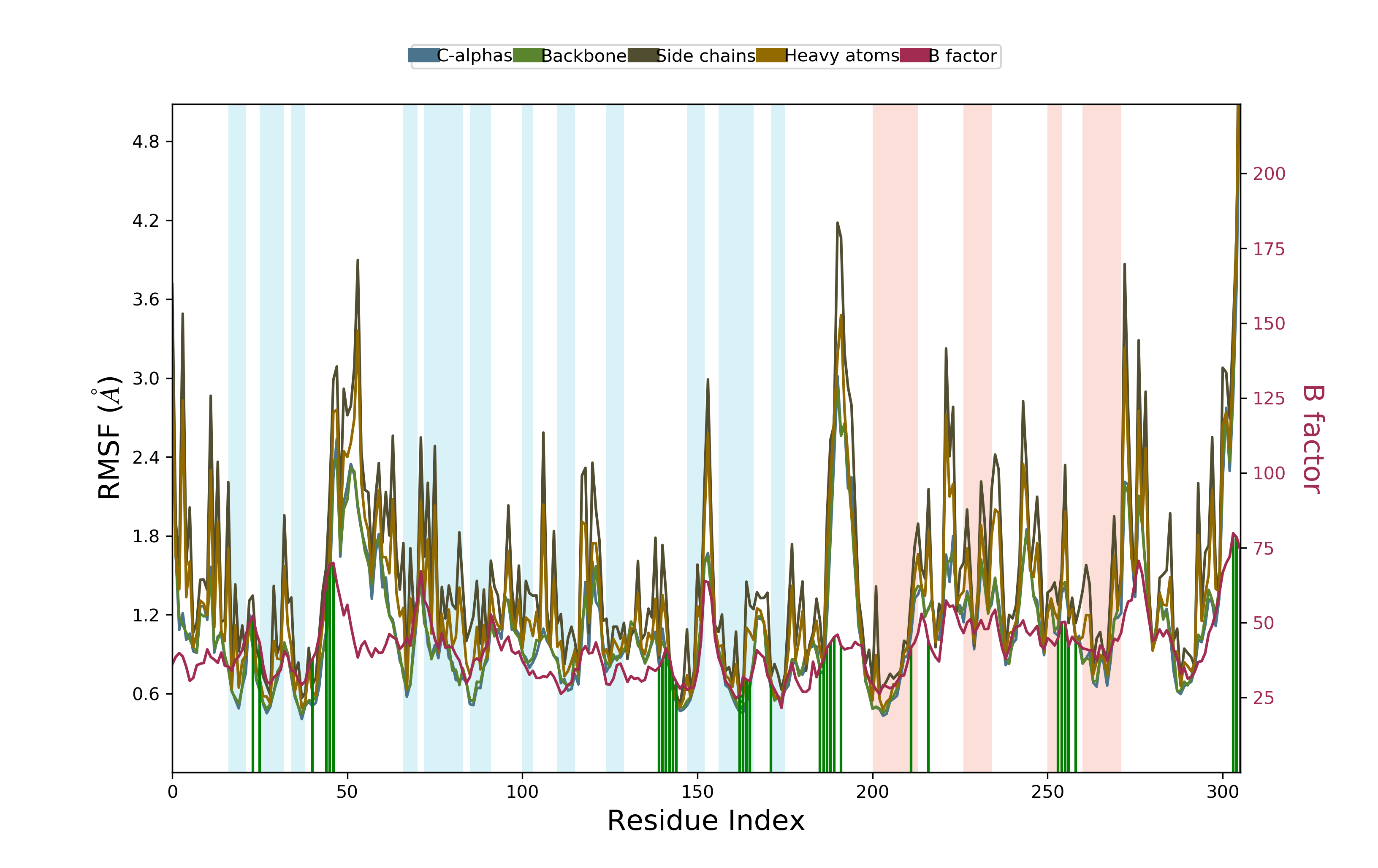


**Fig-S6.** RMSF plot of SARS-CoV-2 M^Pro^ target in complex with Withanoside V. The green bar plotted in the protein residue index corresponds to highly fluctuating amino acid residues.


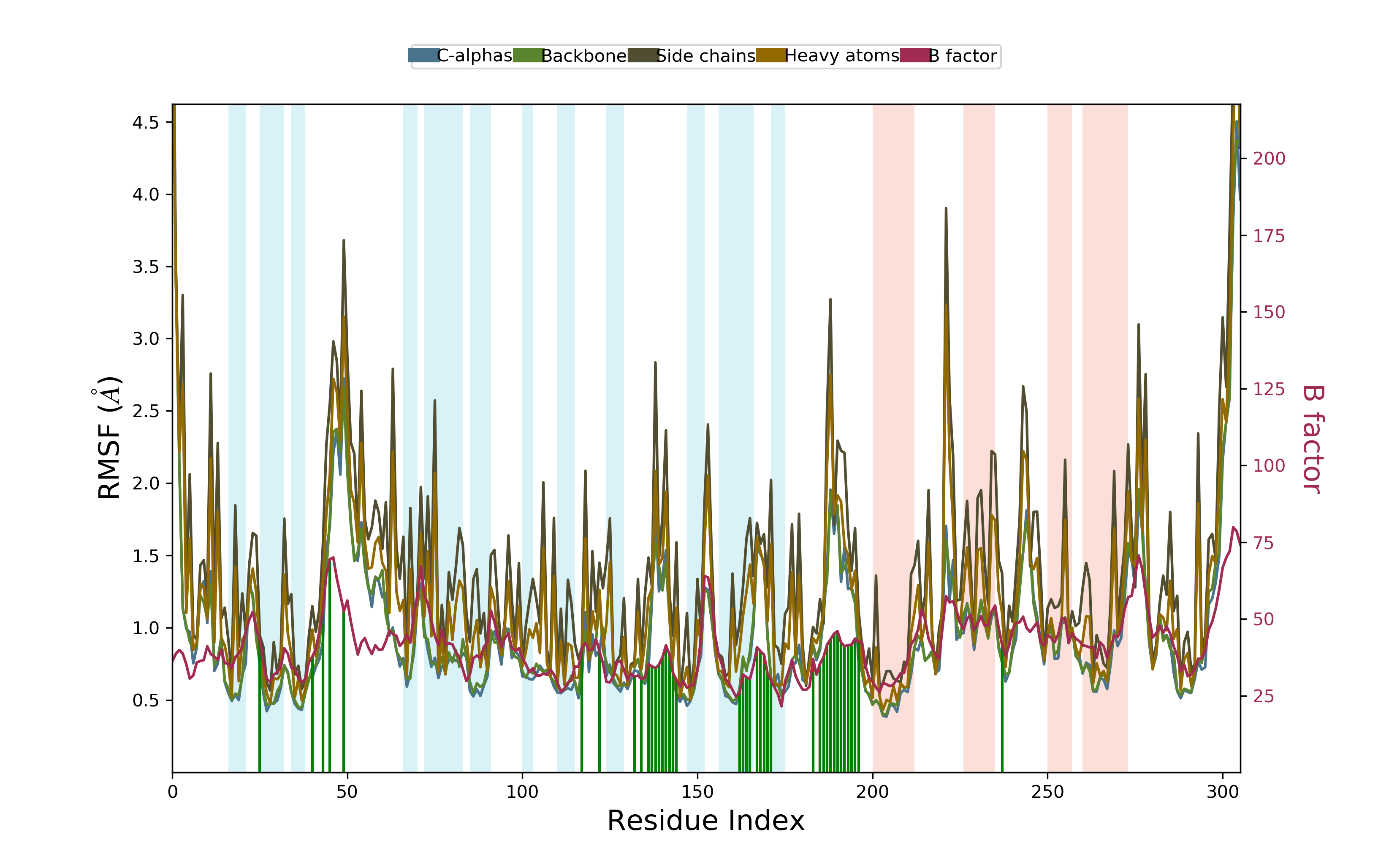


**Fig-S7.** RMSF plot of SARS-CoV-2 M^Pro^ target in complex with Withanoside VI. The green bar plotted in the protein residue index corresponds to highly fluctuating amino acid residues.


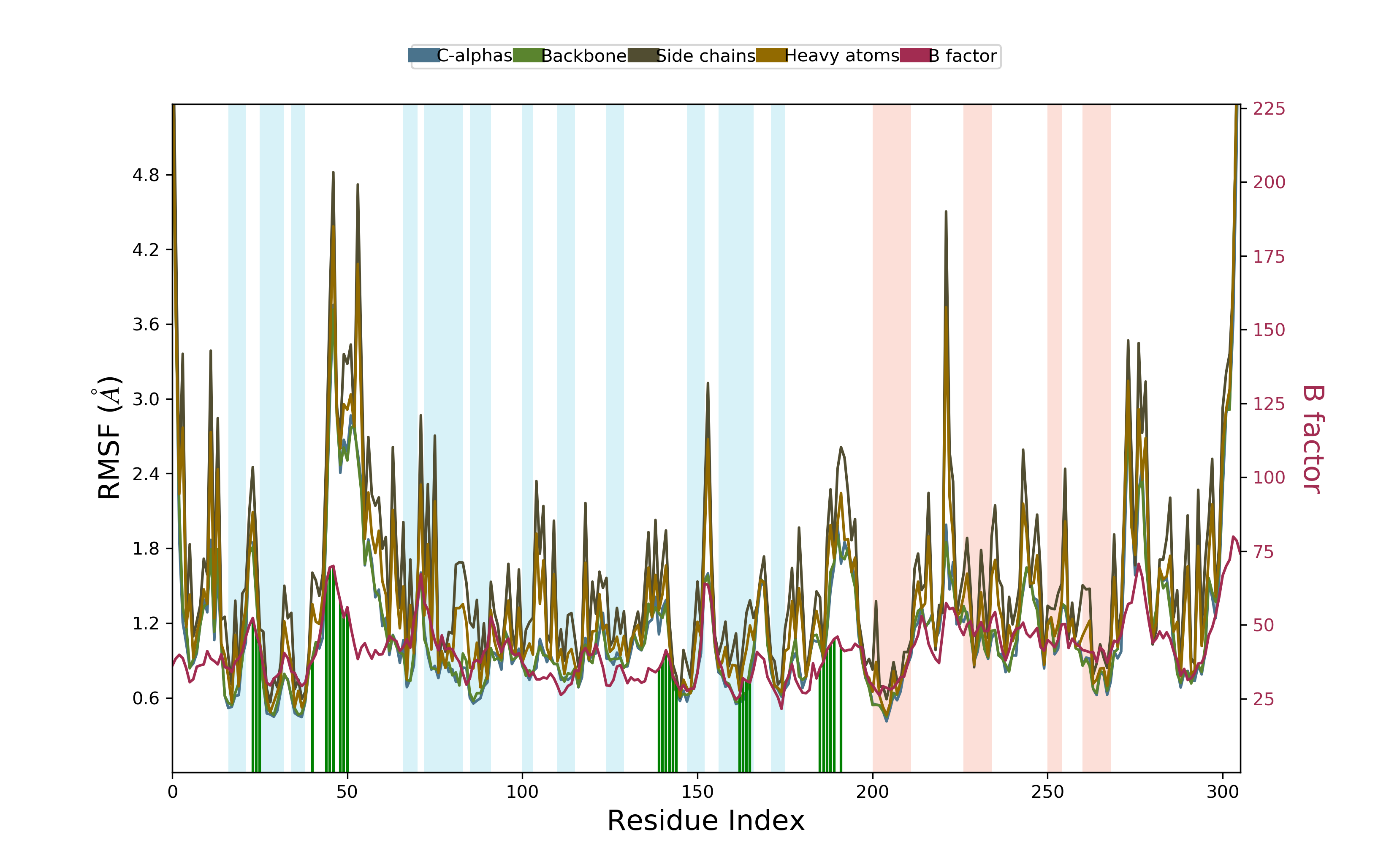


**Fig-S8.** RMSF plot of SARS-CoV-2 M^Pro^ target in complex with Racemoside B. The green bar plotted in the protein residue index corresponds to highly fluctuating amino acid residues.


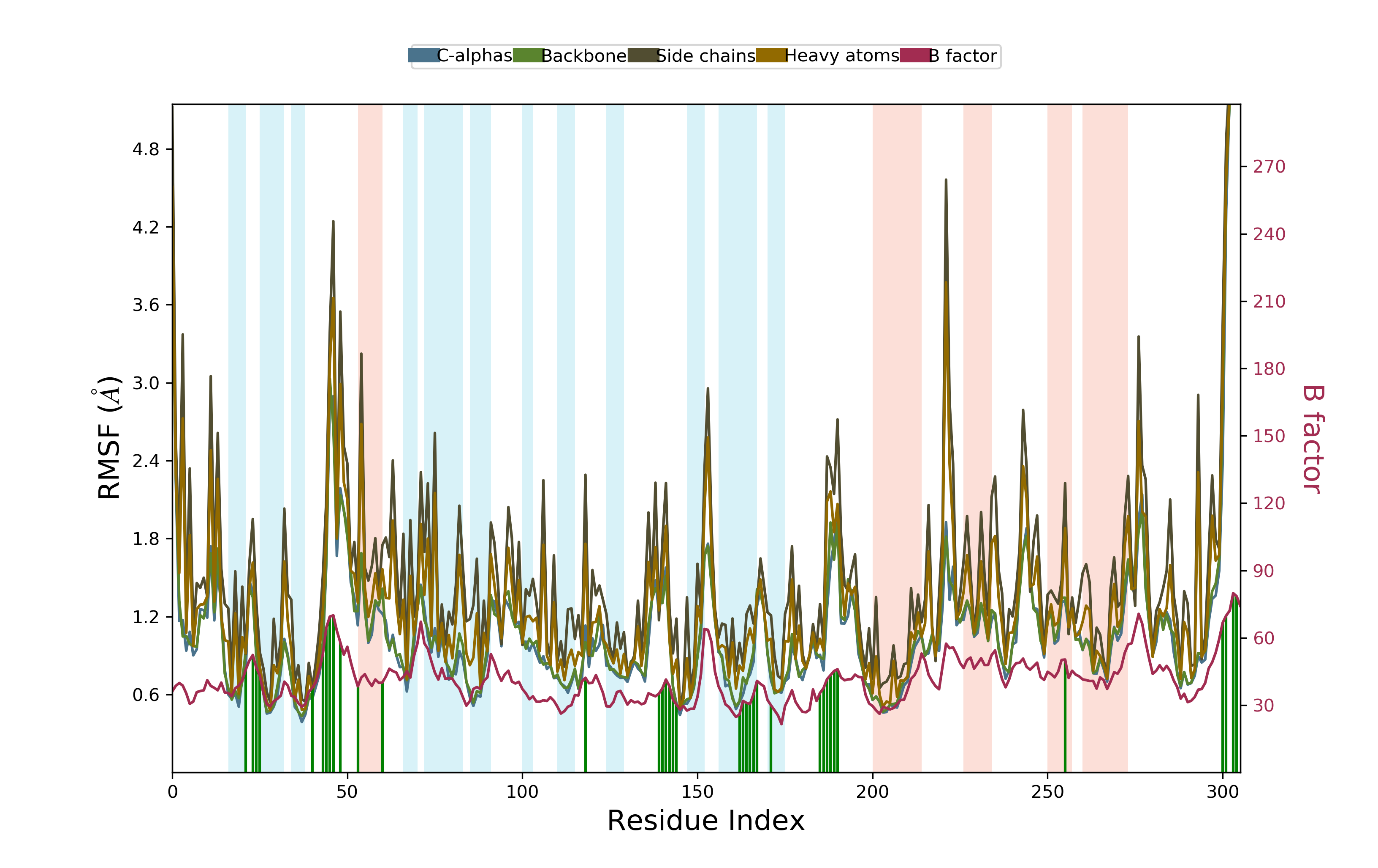


**Fig-S9.** RMSF plot of SARS-CoV-2 M^Pro^ target in complex with Racemoside A. The green bar plotted in the protein residue index corresponds to highly fluctuating amino acid residues.


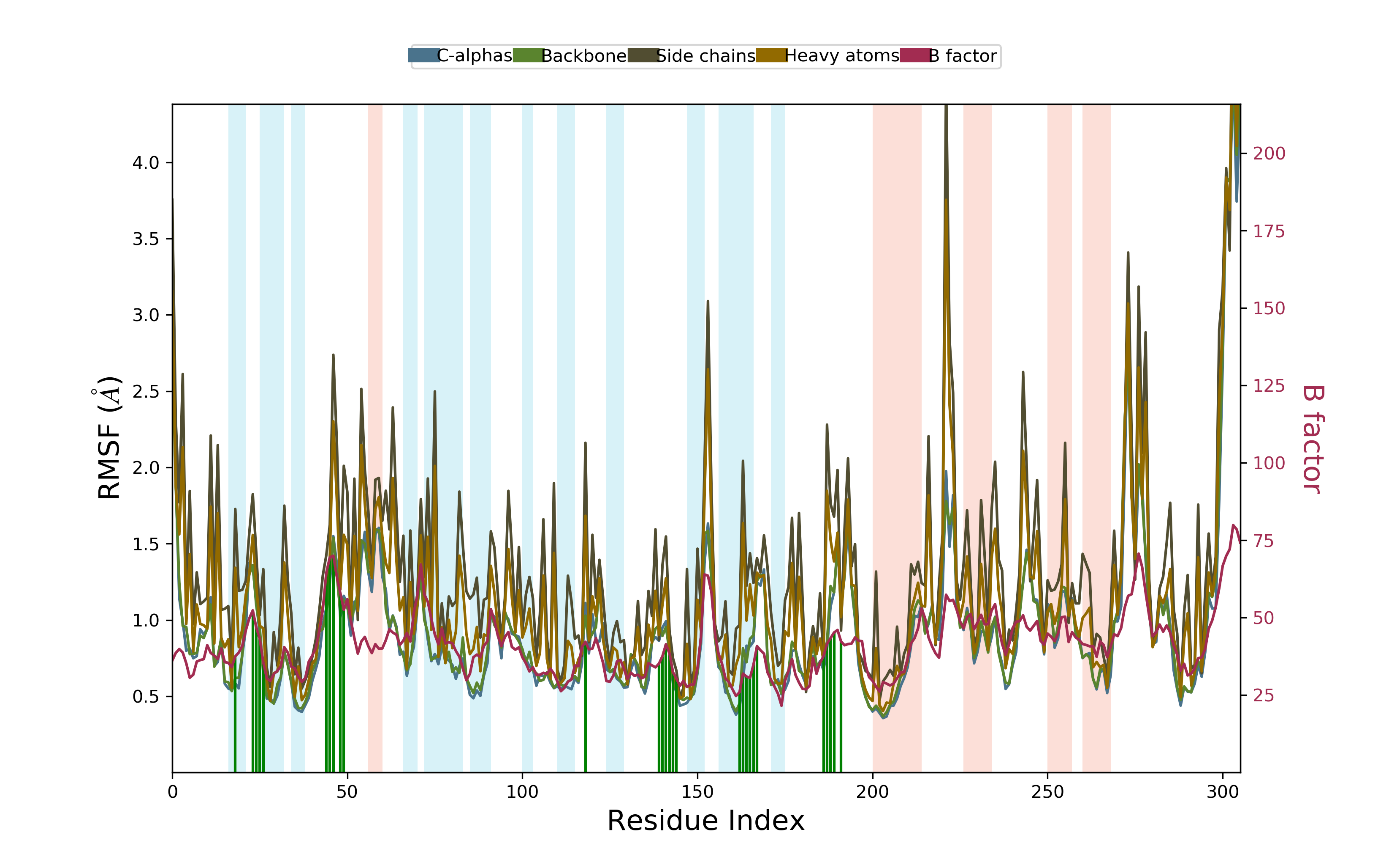


**Fig-S10.** RMSF plot of SARS-CoV-2 M^Pro^ target in complex with Shatavarin IX. The green bar plotted in the protein residue index corresponds to highly fluctuating amino acid residues.


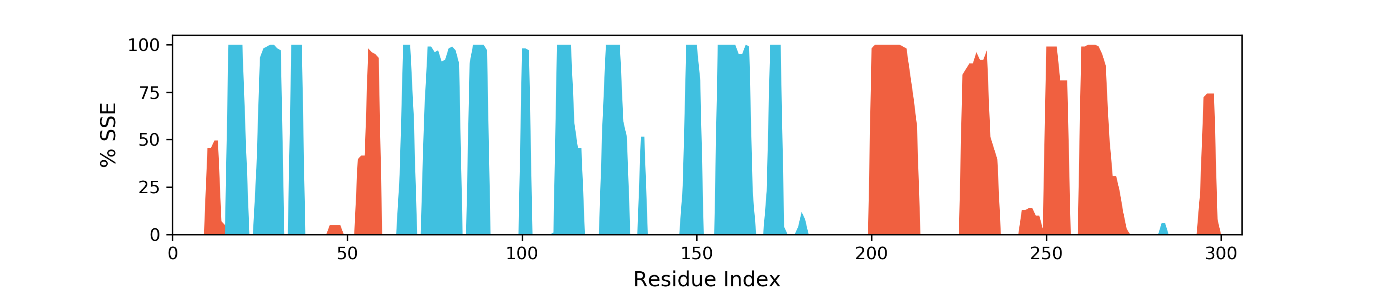


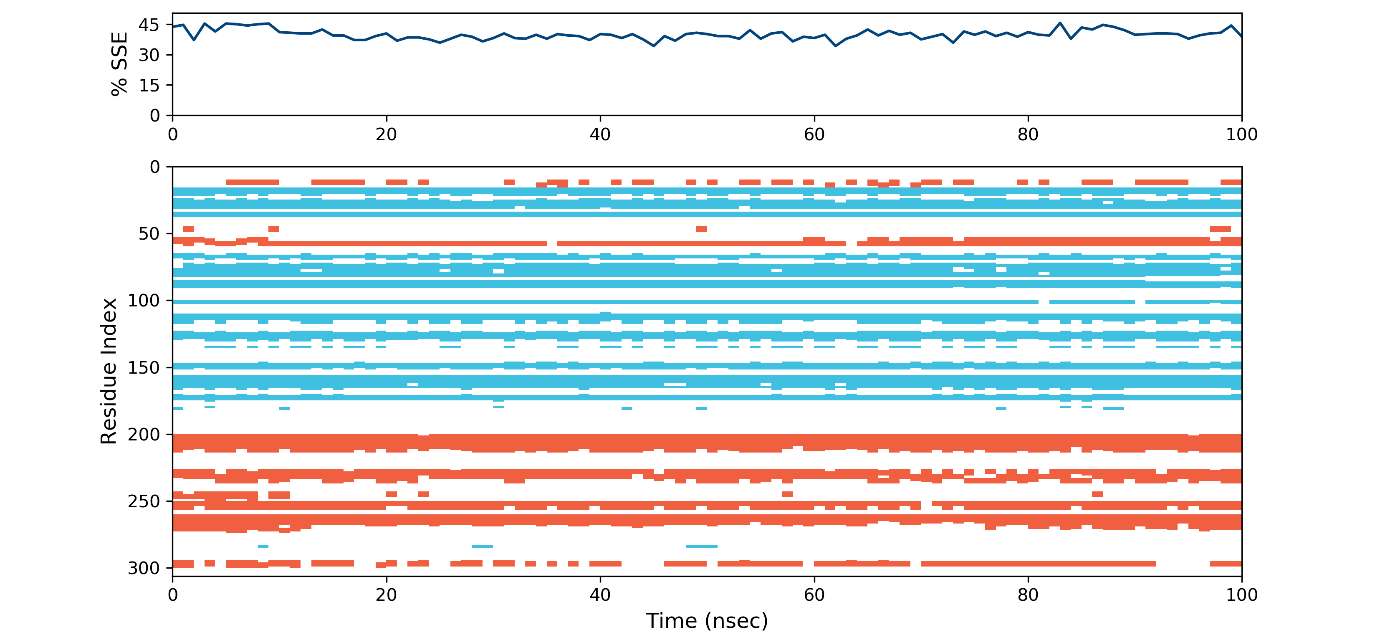


**Fig-S11.** The distribution of secondary structure elements of SARS-CoV-2 M^Pro^ target as a function of simulation time with co-crystal ligand, N3. The red and cyan colors represent helices and beta-sheets, respectively.


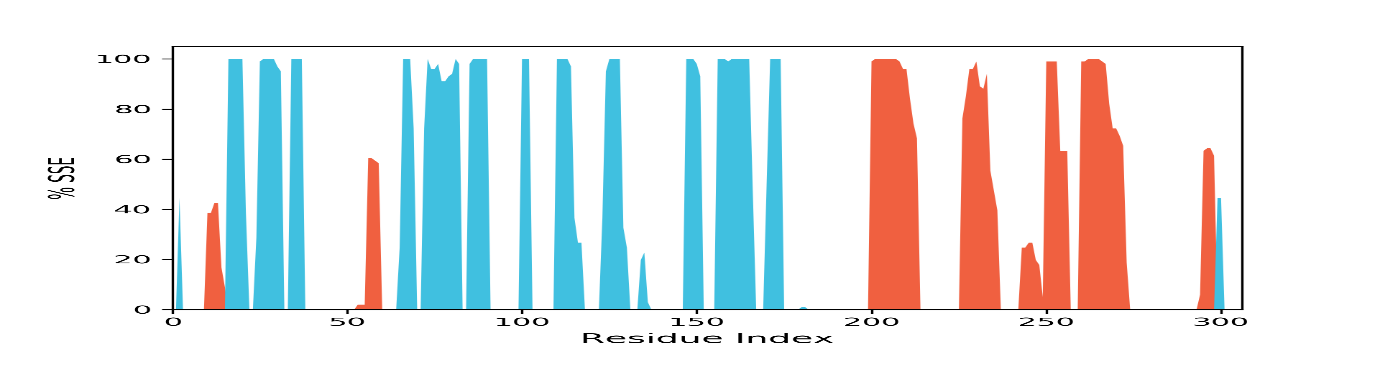


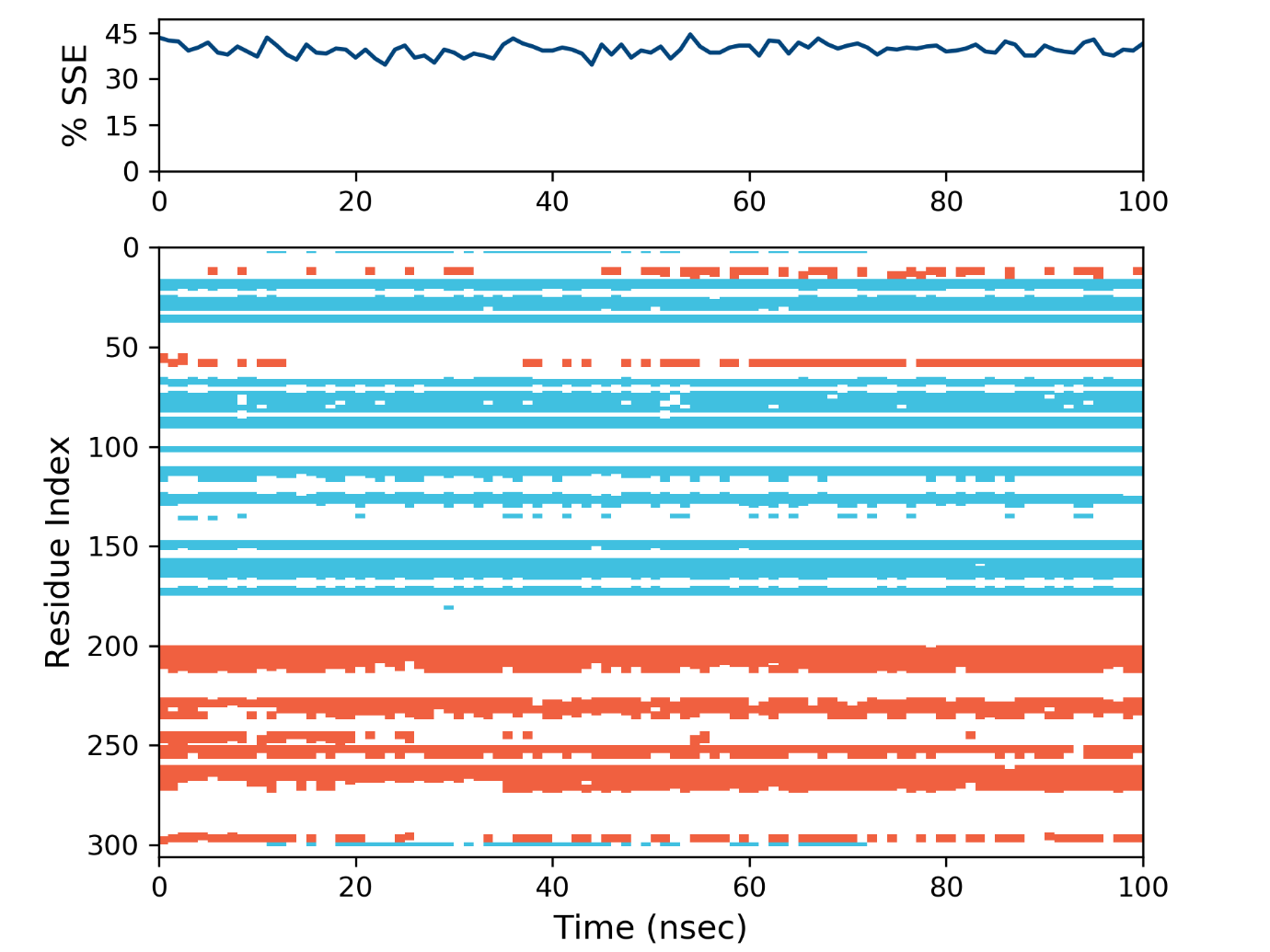


**Fig-S12.** The distribution of secondary structure elements of SARS-CoV-2 M^Pro^ target as a function of simulation time with Withanoside V. The red and cyan colors represent helices and beta-sheets, respectively.


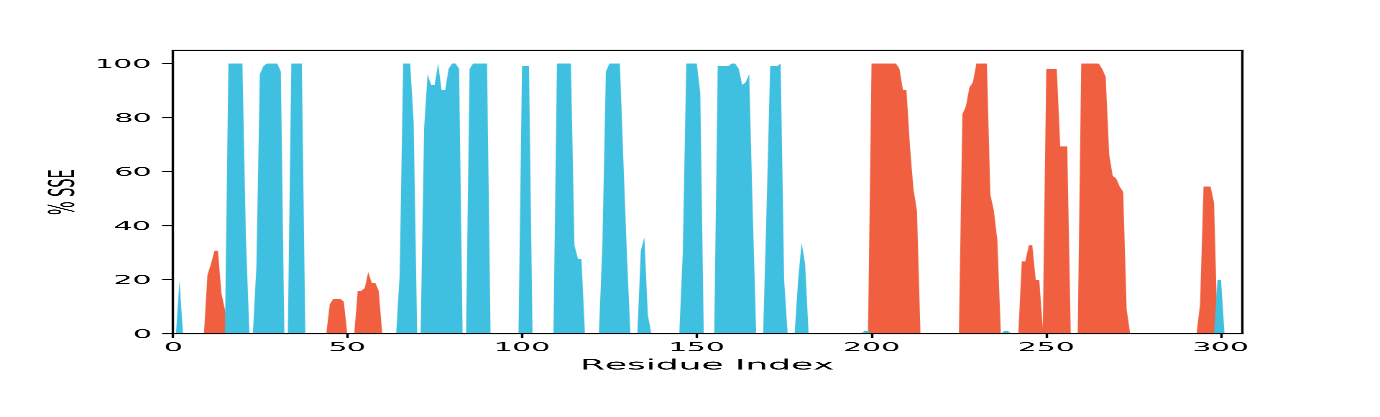


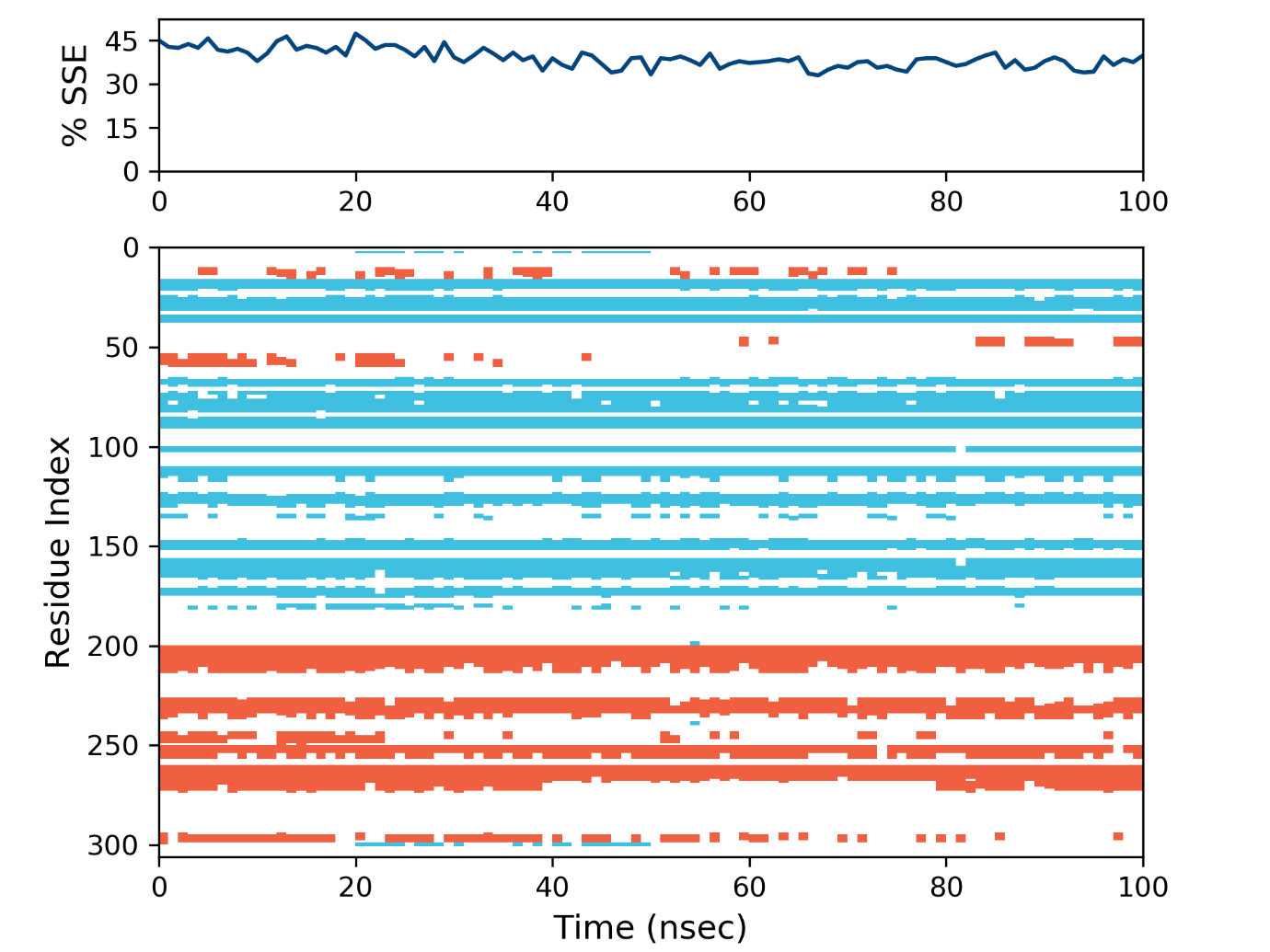


**Fig-S13.** The distribution of secondary structure elements of SARS-CoV-2 M^Pro^ target as a function of simulation time with Withanoside VI. The red and cyan colors represent helices and beta-sheets, respectively.


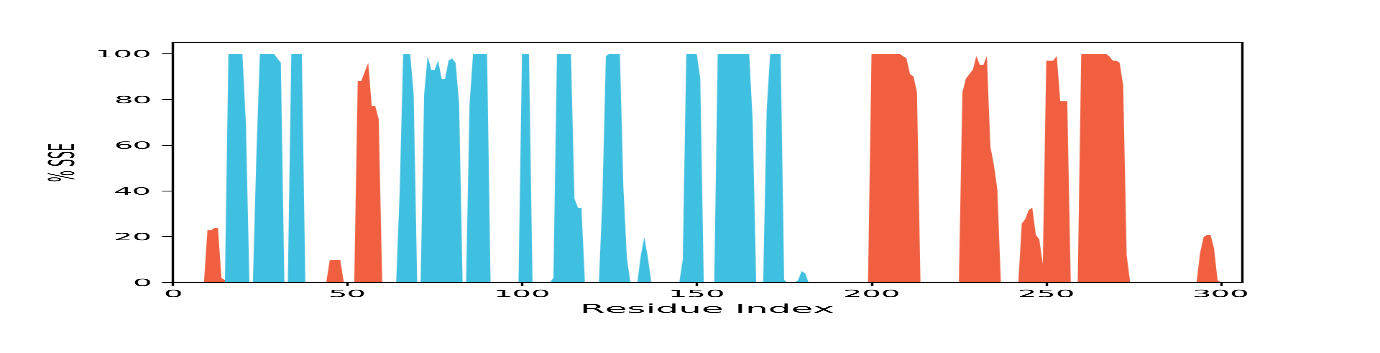


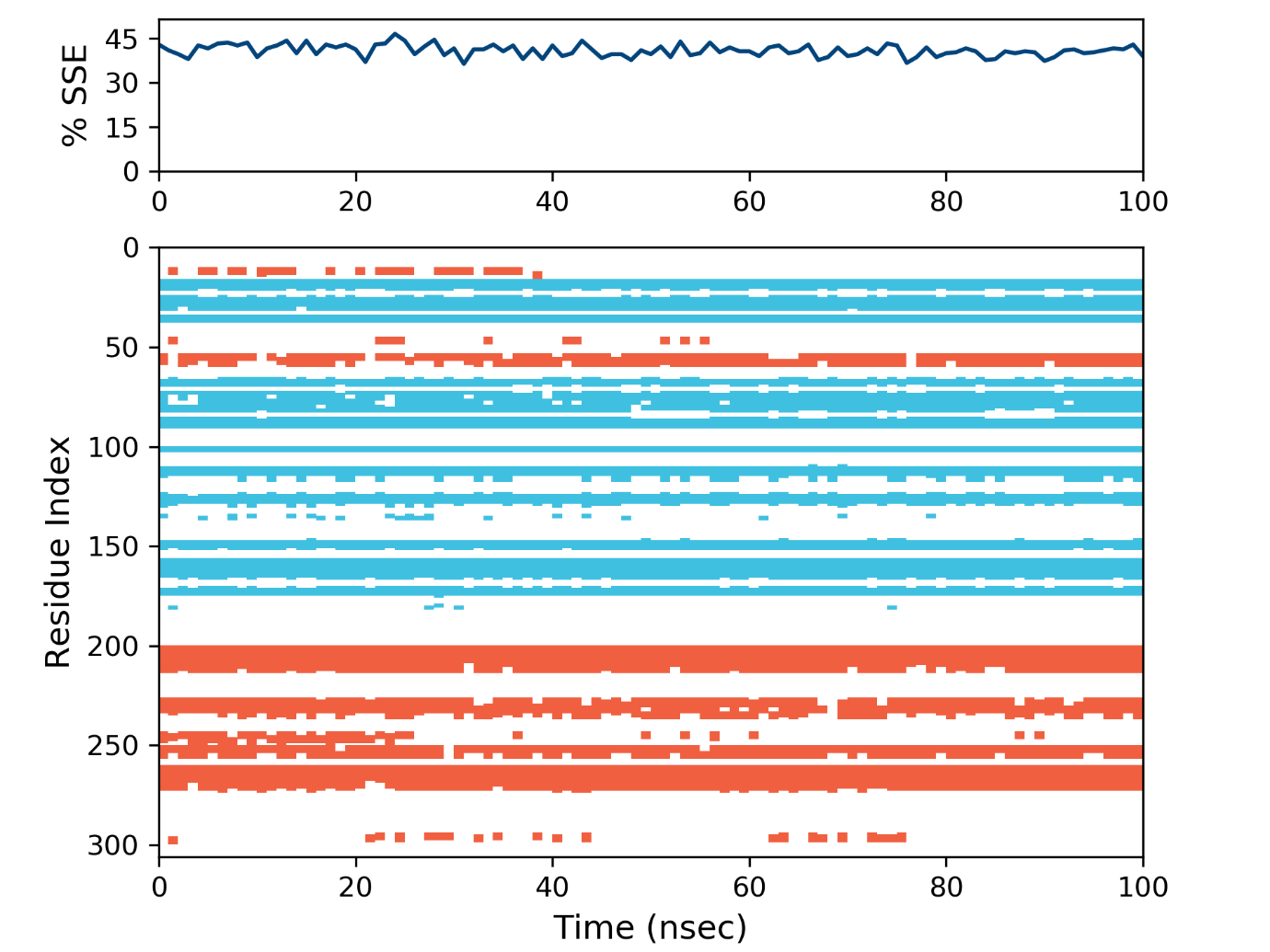


**Fig-S14.** The distribution of secondary structure elements of SARS-CoV-2 M^Pro^ target as a function of simulation time with Racemoside B. The red and cyan colors represent helices and beta-sheets, respectively.


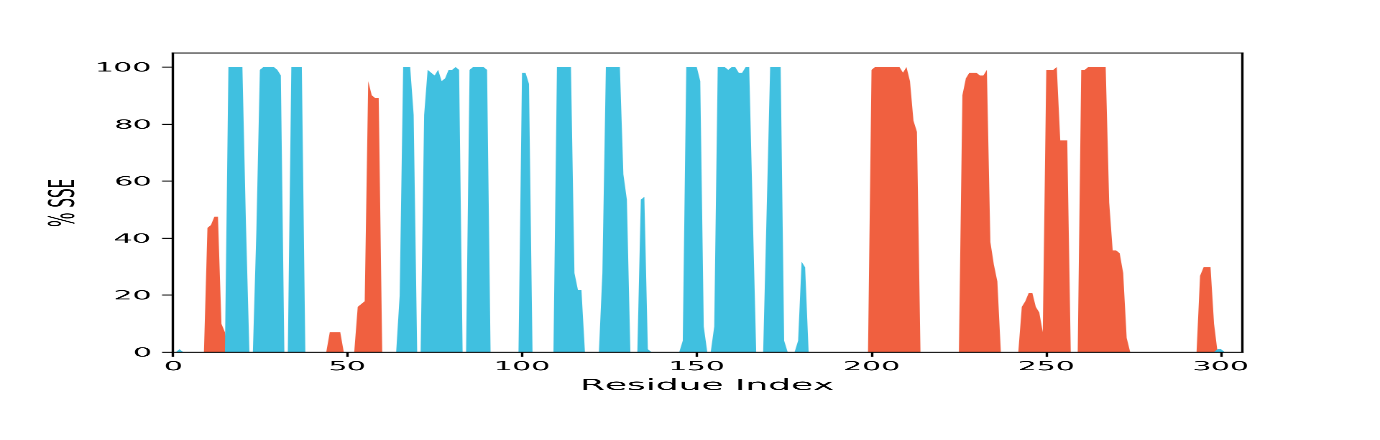


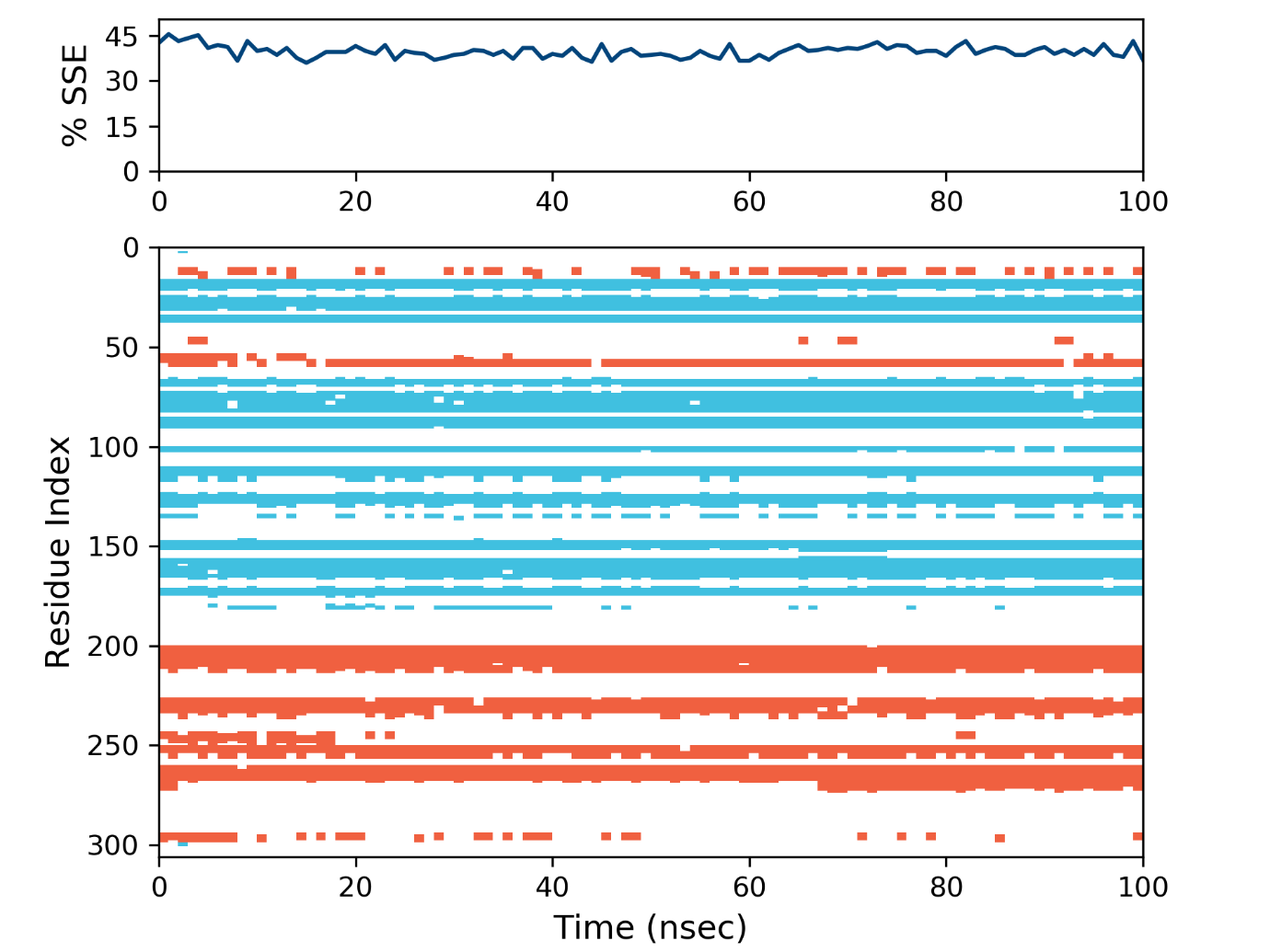


**Fig-S15.** The distribution of secondary structure elements of SARS-CoV-2 M^Pro^ target as a function of simulation time with Racemoside A. The red and cyan colors represent helices and beta-sheets, respectively.


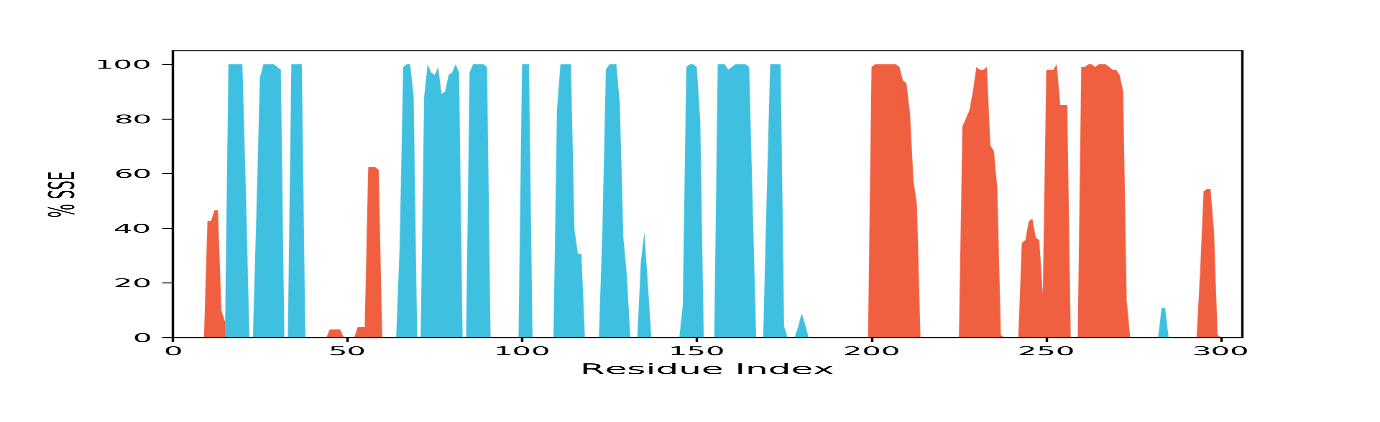


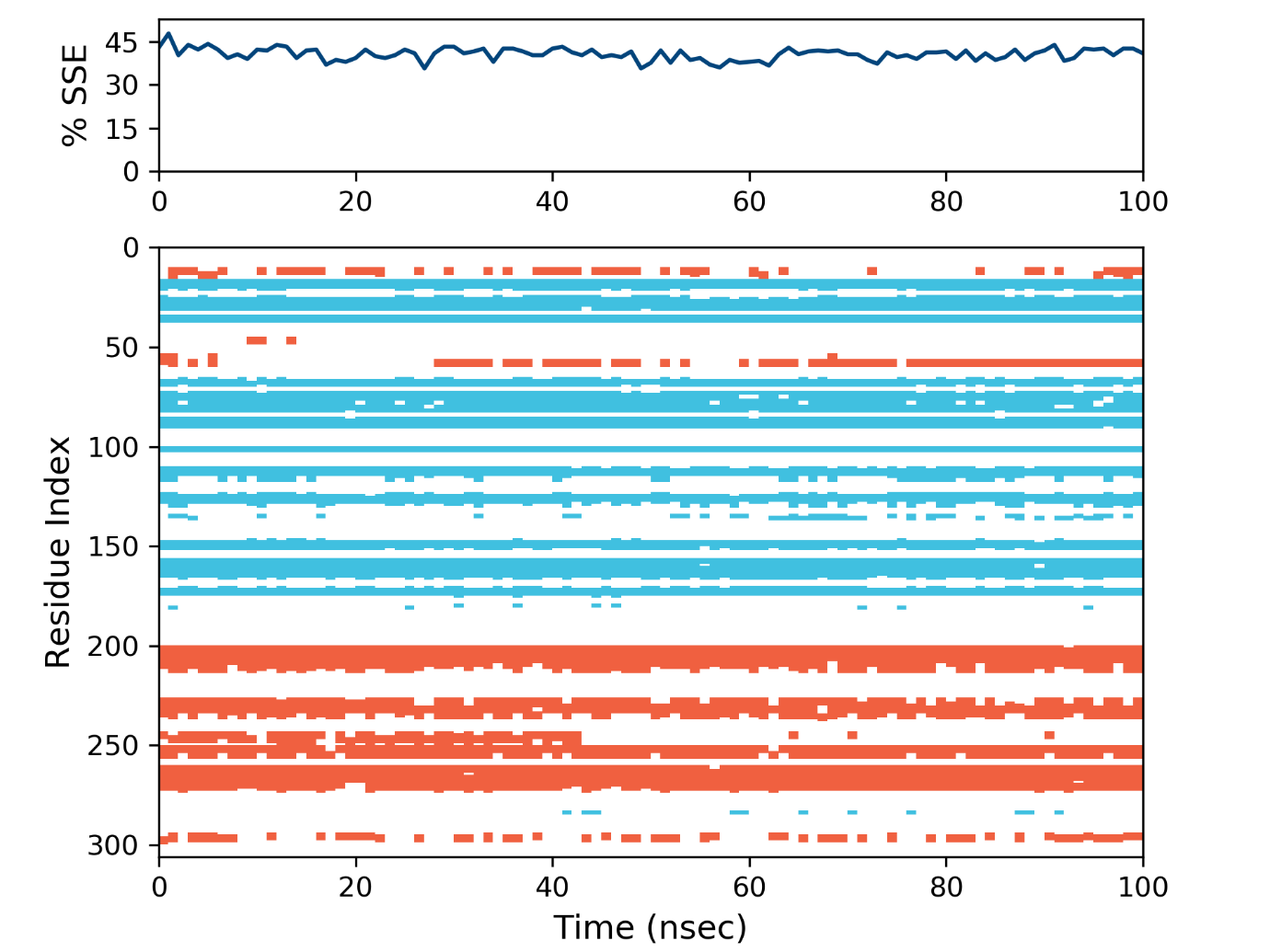


**Fig-S16.** The distribution of secondary structure elements of SARS-CoV-2 M^Pro^ target as a function of simulation time with Shatavarin IX. The red and cyan colors represent helices and beta-sheets, respectively.
